# Natural Killer Group 2D Ligand Depletion Reconstitutes Natural Killer Cell Immunosurveillance of Head and Neck Squamous Cell Carcinoma

**DOI:** 10.3389/fimmu.2017.00387

**Published:** 2017-04-10

**Authors:** Sandra Weil, Stefanie Memmer, Axel Lechner, Volker Huppert, Ariane Giannattasio, Tamara Becker, Andreas Müller-Runte, Karen Lampe, Dirk Beutner, Alexander Quaas, Ralf Schubert, Eva Herrmann, Alexander Steinle, Ulrike Koehl, Lutz Walter, Michael S. von Bergwelt-Baildon, Joachim Koch

**Affiliations:** ^1^NK Cell Biology, Georg-Speyer-Haus, Institute for Tumor Biology and Experimental Therapy, Frankfurt am Main, Germany; ^2^Institute of Medical Microbiology and Hygiene, University of Mainz Medical Center, Mainz, Germany; ^3^Medical Faculty, Department of Otorhinolaryngology, Head and Neck Surgery, University of Cologne, Center for Integrated Oncology Köln Bonn, Cologne, Germany; ^4^Miltenyi Biotec, Bergisch Gladbach, Germany; ^5^Primate Husbandry, German Primate Center, Leibniz Institute for Primate Research, Göttingen, Germany; ^6^Infectious Pathology Unit, German Primate Center, Leibniz Institute for Primate Research, Göttingen, Germany; ^7^Institute of Pathology, University of Cologne, Cologne, Germany; ^8^Allergy, Pulmonology, and Cystic Fibrosis, Children’s Hospital, Goethe University, Frankfurt am Main, Germany; ^9^Institute for Biostatistics and Mathematical Modelling, Goethe University, Frankfurt am Main, Germany; ^10^Institute for Molecular Medicine, Goethe University, Frankfurt am Main, Germany; ^11^Hannover Medical School, Institute for Cellular Therapeutics, IFB-Tx, Hannover, Germany; ^12^Primate Genetics Laboratory, German Primate Center, Leibniz Institute for Primate Research, Göttingen, Germany; ^13^Cologne Interventional Immunology Department I of Internal Medicine, University Hospital of Cologne, Cologne, Germany

**Keywords:** adsorption apheresis, head and neck squamous cell carcinoma, immunotherapy of cancer, natural killer cells, natural killer group 2D ligands, tumor immune escape

## Abstract

Head and neck squamous cell carcinoma (HNSCC) is a highly heterogeneous and aggressive tumor originating from the epithelial lining of the upper aero-digestive tract accounting for 300,000 annual deaths worldwide due to failure of current therapies. The natural killer group 2D (NKG2D) receptors on natural killer (NK) cells and several T cell subsets play an important role for immunosurveillance of HNSCC and are thus targeted by tumor immune evasion strategies in particular by shedding of various NKG2D ligands (NKG2DLs). Based on plasma and tumor samples of 44 HNSCC patients, we found that despite compositional heterogeneity the total plasma level of NKG2DLs correlates with NK cell inhibition and disease progression. Strikingly, based on tumor spheroids and primary tumors of HNSCC patients, we found that NK cells failed to infiltrate HNSCC tumors in the presence of high levels of NKG2DLs, demonstrating a novel mechanism of NKG2DL-dependent tumor immune escape. Therefore, the diagnostic acquisition of the plasma level of all NKG2DLs might be instrumental for prognosis and to decipher a patient cohort, which could benefit from restoration of NKG2D-dependent tumor immunosurveillance. Along these lines, we could show that removal of shed NKG2DLs (sNKG2DLs) from HNSCC patients’ plasma restored NK cell function *in vitro* and in individual patients following surgical removal of the primary tumor. In order to translate these findings into a therapeutic setting, we performed a proof-of-concept study to test the efficacy of adsorption apheresis of sNKG2DLs from plasma after infusion of human MICA in rhesus monkeys. Complete removal of MICA was achieved after three plasma volume exchanges. Therefore, we propose adsorption apheresis of sNKG2DLs as a future preconditioning strategy to improve the efficacy of autologous and adoptively transferred immune cells in cellular cancer immunotherapy.

## Introduction

Head and neck squamous cell carcinoma (HNSCC) is a highly aggressive solid tumor originating from the epithelial lining of the upper aero-digestive tract and is characterized by phenotypic, etiological, biological, and clinical heterogeneity (1, 2). With approximately 650,000 newly diagnosed patients and 300,000 deaths annually, HNSCC is the sixth most common cancer worldwide (3, 4). The relative frequency of HNSCC correlates with tobacco and alcohol consumption (5) and human papillomavirus (HPV) infection (6–8). Current therapeutic regimens combine surgery, radiotherapy and chemotherapy with 5-year survival rates of 30–65 and 5–58% for the tumor stages T1–T4 and N0–N3, respectively (9, 10). However, conventional therapies are associated with high morbidity and toxicity and have mainly palliative benefits for patients with metastatic disease or incurable recurrence (11, 12).

Human natural killer (NK) cells are innate lymphoid effector cells which can be divided into two subsets according to their cell surface expression levels of CD56 and CD16. CD56^dim^/CD16^bright^ NK cells predominantly mediate natural cytotoxicity, whereas the CD56^bright^/CD16^dim^ subset plays a role in immune regulation through a high cytokine secretion potential (13, 14). NK cells mediate immunosurveillance of malignantly transformed cells (15, 16). Their cytotoxicity is regulated by a dynamic balance of agonistic and antagonistic signaling from activating and inhibitory receptors (16). Among the major activating receptors is the natural killer group 2D (NKG2D) receptor (17–19), which is also expressed on T cell subtypes and NKT cells (20). NKG2D recognizes the structurally related ligands (NKG2DLs) MHC class I chain-related protein A (MICA), MICB, and several UL-16-binding proteins commonly overexpressed on tumor cells (21–23). However, shedding of NKG2DLs leads to impaired cytotoxicity of NK cells and T cells due to loss of antigenic NKG2DLs and shed NKG2DL (sNKG2DL)-induced downregulation of NKG2D (24–26). Consequently, patients suffering from various cancers (27–33) demonstrated an inverse correlation of sMICA plasma levels and NKG2D-dependent NK cell cytotoxicity. Since all sNKG2DLs engage with a common receptor, the cumulative level of all sNKG2DLs is decisive for their biological/clinical effect. However, the majority of studies so far investigated only individual sNKG2DLs.

Cellular immunotherapy based on autologous or adoptive NK or T cells, including adoptive chimeric antigen receptor (CAR)-functionalized cells, represents a novel therapeutic concept for cancer treatment (34, 35). All of these cells might be affected by sNKG2DLs in the same way than the patients’ endogenous NKG2D^+^ immune cells thus limiting therapeutic efficacy. Therefore, the current study aimed at molecular characterization of NKG2D-dependent immune escape and development of a procedure to restore NKG2D-dependent immunosurveillance in HNSCC patients.

## Materials and Methods

### Patients

Plasma from 44 HNSCC patients (mean age: 65 years) and 12 age-matched healthy volunteers (mean age: 55 years) was analyzed. Patients’ demographics and tumor characteristics are summarized in Table 1. Tumor staging was assigned according to the TNM classification of the International Union Against Cancer. Thirty of the HNSCC patients were studied at primary diagnosis and 14 patients with recurrent/metastatic (relapse) disease. From the patients with primary diagnosis, blood samples of 26 patients were taken before therapeutic treatment (treatment naïve), while 4 patients had surgery and/or radiotherapy before sample collection. Ten patients with recurrent disease were under palliative therapy during sample collection, while four patients were currently not under therapy.

**Table 1:** **Clinical parameters and frequencies of head and neck squamous cell carcinoma patients**.

Parameter	Absolute numbers and frequency (%)
**Age (mean: 65 years)**	
20–40	1 (2.3)
40–60	15 (34.1)
60–80	24 (54.5)
>80	4 (9.1)
**Sex**	
Male	39 (88.6)
Female	5 (11.4)
**Disease stage**	
I	3 (6.8)
II	4 (9.1)
III	7 (15.9)
IVA	19 (43.2)
IVB	2 (4.5)
IVC	6 (13.6)
Other	3 (6.8)
**Localization of tumor**	
Oral cavity	10 (22.7)
Oropharynx	9 (20.5)
Hypopharynx	12 (27.3)
Larynx	7 (15.9)
Larynx/hypopharynx	3 (6.8)
Other	3 (6.8)
**Therapy**	
No therapy	30 (68.2)
Post surgery	2 (4.5)
Post surgery and RCTx	2 (4.5)
Palliative RTx/CTx	10 (22.7)
**Status**	
Primary	30 (68.2)
Recurrent (relapse)	14 (31.8)
**TNM classification**	
T	
1	5 (11.4)
2	8 (18.2)
3	9 (20.5)
3–4/4	17 (38.6)
n.d.	5 (11.4)
N	
0	16 (36.4)
1	6 (13.6)
2	1 (2.3)
2b	9 (20.5)
2c	7 (15.9)
3	3 (6.8)
n.d.	2 (4.5)
M	
0	38 (86.4)
1	6 (13.6)
**Human papillomavirus status**	
Positive	4 (9.1)
Negative	34 (77.3)
n.d.	6 (13.6)
**Noxa**	
None	11 (25.0)
Smoking	16 (36.4)
Alcohol	1 (2.3)
Smoking + alcohol	16 (36.4)

### Animal Experiments

Two male rhesus macaques (age 6 years) from the German Primate Center breeding colony (German Primate Center, Göttingen) were used for the *in vivo* experiments (pilot study and apheresis). All experimental procedures were done under inhalation anesthesia. The animals were i.v. injected with sMICA*04 at 100 µg/l blood volume (blood volume corresponds to approximately 7% of body weight). Plasma volume was calculated based on individual hematocrit. For the apheresis, animals were connected to a Life18™ apheresis unit equipped with an adsorber cartridge (anti-MICA antibody covalently coupled to sepharose Cl-4B at 0.95 mg AMO1/g sepharose) *via* a double lumen catheter in the *vena femoralis*. Apheresis was started 15 min post-injection with a flow-rate of 25–30 ml/min until three complete plasma volume exchanges were reached. During apheresis, animals got heparin as anticoagulant (50 U/kg). Blood and urine samples were taken every 15 min.

### Cell Culture

Primary NK cells were purified (>95% pure) from buffy coats of healthy volunteers kindly provided by the German Red Cross Blood Service (Institute for Transfusion Medicine and Immunohematology, Medical School, Goethe University Frankfurt, Germany) with written donor approval and approval by the Ethics Committee of the Goethe University Frankfurt (Frankfurt am Main, Germany, permit #329/10). Therefore, peripheral blood mononuclear cells (PBMCs) were isolated by a density gradient with Biocoll (Biozol, Germany) followed by untouched NK cell isolation using magnetic cell separation with the human NK cell isolation kit (Miltenyi Biotec, Germany). NK cells were expanded and activated for 7 days in X-Vivo10 medium (Lonza, Switzerland) supplemented with 5% human serum (Life Technologies, USA), 1,000 IU/ml IL-2 (Promokine, Germany), and NK cell activation/expansion kit (Miltenyi Biotec, Germany). The cell lines FaDu (HTB-43, human pharynx squamous cell carcinoma cells), CAL27 (CRL-2095, human tongue squamous cell carcinoma cells), and Detroit562 (CCL-138, human pharyngeal carcinoma cells) were purchased from the American Type Culture Collection. HNSCC cell lines were cultured in MEM medium (Life Technologies, USA). SiHa (grade II, human cervix squamous cell carcinoma) cells kindly provided by Dr. Adelheid Cerwenka (DKFZ, Heidelberg, Germany) were cultured in DMEM medium (Life Technologies, USA). All media were supplemented with 10% FCS (PAA and PAN Biotech, Germany), 1% penicillin/streptomycin (Life Technologies, USA), 2 mM l-glutamine (Life Technologies, USA), and 1 mM sodium pyruvate for HNSCC cell lines (Life Technologies, USA).

### Cytokine Measurement with Cytometric Bead Array (CBA)

The concentrations of IFNγ, TNFα, IL-1, IL-6, IL-8, IL-10, MCP-1, RANTES, MIP-1α, MIP-1β, and TGF-β1 in plasma of HNSCC patients and healthy donors were determined using the BD™ CBA Flex Set System (BD Bioscience, USA), according to manufacturer’s instructions. Samples were diluted 1:30 for TGF-β1 or 1:4 for all other sets and tested in duplicates. Samples were measured with the BD FACS Array™ and analyzed with FCAP Array Software (BD Bioscience, USA). Cytokine levels of HNSCC patient samples were normalized to healthy controls, and data are represented as *x*-times median control.

### ELISA

For analyses of sNKG2DL levels in the plasma of HNSCC patients, healthy donors, rhesus monkeys, or tumor spheroid supernatants [concentrated 10-fold using Amicon centrifugal filter units (Merckmillipore, Germany)], samples were diluted 1:2 and measured in duplicates. Soluble NKG2DLs were analyzed as described (27, 36–38). Briefly, ELISA plates (Greiner Bio-One, Austria) were coated with mouse anti-human MICA (clone AMO1), anti-human MICA/B (clone BAMO1), anti-human ULBP1 (clone AUMO5), anti-human ULBP2 (clone BUMO1), or goat anti-human ULBP3 antibodies (AF1517; R&D Systems, USA). After saturation with BSA blocking solution (Candor, Germany), the plates were incubated with soluble MICA*04 or recombinant Fc-fusion proteins of human MICB, ULBP1/2/3 (1599-MB-050, 1380-UL-050, 1298-UL-050, 1517-UL-050; R&D Systems, USA) as standards. For detection and quantification of soluble ligands, mouse anti-human MICA/B (clone BAMO3), anti-human MICB (clone BMO2), anti-human ULBP1 (clone AUMO2), or anti-human ULBP2 (MAB1298; R&D Systems, USA) antibodies were used in combination with a goat anti-mouse IgG2a horseradish peroxidase (HRP)-conjugated antibody (1080-05; Southern Biotechnologies, USA). For sULBP3, the mouse anti-human ULBP3 antibody (clone CUMO3) was used in combination with a goat anti-mouse IgG1 HRP-conjugated antibody (1070-05; Southern Biotechnologies, USA). Following visualization with 3,3′,5,5′-tetramethylbenzidine substrate (KPL, Germany), signals were measured in a microtiter plate reader (λ = 450 nm) and analyzed with Prism 6 software (GraphPad, USA). Data of tumor spheroids were calculated as mean ± SEM per 10,000 cells (per spheroid) of three independent experiments (*n* = 3). Data of plasma samples were calculated as mean ± SEM. Statistical analysis was performed using one-way analysis of variance (ANOVA).

### Depletion of sNKG2D Ligands Using Antibody-Functionalized Beads

Soluble NKG2DLs were depleted from plasma of HNSCC patients, healthy donors, or CAL27 cell culture supernatants (collected on d3 of culture and 10× concentrated). Samples were incubated with a cocktail of Protein A Dynabeads (Life Technologies, USA) coupled to anti-human MICA (clone AMO1), anti-human MICB (MAB1599), anti-human ULBP1 (MAB1380), anti-human ULBP2 (MAB1298), or anti-human ULBP3 (MAB1517; all R&D Systems, USA) antibodies.

For the depletion of sMICA, the anti-MICA antibody (clone AMO1) was covalently coupled to MACSxpress beads (Miltenyi Biotec, Germany). Human plasma of healthy donors supplemented with 20 ng/ml sMICA*04 or culture supernatants of C1R cells transfected with MICA*01/04/07 and MICA*08 (38) were incubated with antibody-coupled beads overnight. Beads and bound sNKG2DLs were removed from the supernatants by magnetic bead separation on a MACSxpress separator (Miltenyi Biotec, Germany) following centrifugation.

### Cytotoxicity Assays

Primary human NK cells of different healthy donors were pre-incubated overnight with sNKG2DL-depleted or non-depleted plasma of HNSCC patients or healthy donors’ plasma as controls. SiHa target cells were labeled with carboxyfluorescein succinimidyl ester (CFSE; Life Technologies, USA) according to manufacturer’s instructions. Labeled SiHa target cells and pre-treated NK cells were cocultured at an E:T ratio of 15:1 for 2 h. NK cells were labeled with mouse anti-human CD45-APC antibodies (clone 5B1, Miltenyi Biotec, Germany), and live/dead cell discrimination was achieved by staining with SytoxBlue (Life Technologies, USA) prior to the measurement on a BD FACSCanto II instrument equipped with a 96-well plate high throughput sampler (HTS). Cellular event counts were analyzed with FlowJo software. The percentage of cell lysis was calculated by evaluation of viable (CFSE^+^/SytoxBlue^−^) and dead (CFSE^+^/SytoxBlue^+^) target cells. Data were normalized to mean of healthy control and are represented as mean ± SEM of triplicates of a representative experiment. Statistical significance was determined by one-way ANOVA.

### Tumor Spheroid Assays

Cytotoxicity assays were performed as previously described (39). In brief, CFSE-labeled HNSCC and SiHa solid tumor spheroids were co-incubated with primary human NK cells at appropriate E:T ratios (5:1) in medium without IL-2 for 48 h. For inhibition, NK cells were pre-incubated overnight with non-/sNKG2DL-depleted CAL27 SN. For endpoint analyses, single tumor spheroids were dissociated, NK cells were labeled with a mouse anti-human CD45-APC antibody (clone 5B1; Miltenyi Biotec, Germany), and live/dead cell discrimination was achieved by staining with SytoxBlue (Life Technologies, USA). Complete samples were measured on a FACS Canto II instrument equipped with a HTS and cellular event counts were analyzed with FlowJo software. The percentage of cell lysis was calculated by evaluation of viable (CFSE^+^/SytoxBlue^−^) and dead (CFSE^+^/SytoxBlue^+^) target cells. Data are represented as mean ± SEM of eight spheroids. Statistical significance was determined by one-way ANOVA.

For infiltration studies, tumor cells and CAL27 SN or sMICA*01 pre-treated NK cells were seeded 1:1 into agarose coated 96-well plates. Individual spheroids (*n* > 8) were collected after 48 h and embedded into OCT compound (Sakura). Cryosections (7 µm) of solid tumor spheroids were stained with rabbit anti-human cleaved caspase-3 (clone 5A1E; Cell Signaling Technology, USA) and mouse anti-human CD45 (clone 2B11 + PD7/26; DAKO, Germany) and analyzed by standard two-step polymer methods with Envision kits (DAKO, Germany). 3,3′-diaminobenzidine (DAB) and permanent red or 3-amino-9-ethylcarbazol (AEC) were used as chromogen. Counterstaining was performed using hematoxylin (DAKO, Germany) according to the manufacturer’s instructions. Phase-contrast pictures of solid spheroids were analyzed by triple measurement of independent spheroids (*n* = 8) for each condition by ImageJ software (40). Tumor spheroid volume was calculated based on area analysis of solid spheroids assuming a perfect sphere. The number of infiltrated NK cells was calculated as percentage of CD45^+^ NK cells from whole cell numbers in spheroid cryosections. Data are represented as mean ± SEM of eight tumor spheroid cryosections and statistical analysis was performed using unpaired Student’s two-tailed *t*-test.

### Flow Cytometry

Cryo-preserved PBMCs of healthy volunteers or HNSCC patients and corresponding disintegrated primary tumor samples were stained with a cocktail of mouse anti-human CD3-APC.Cy7 (clone HIT3a), anti-human CD4-PacificBlue (clone OKT4), anti-human CD8a-AlexaFluor700 (clone HIT8a), anti-human CD16-FITC (clone 3G8), anti-human CD56-PE.Cy7 (clone HCD56) (all, BioLegend, USA), anti-human CD45-Pe.Flour610 (clone HI30; eBioscience, USA), and anti-human CD314-PE (clone BAT221; Miltenyi Biotec, Germany). For cell viability, Aqua Dead Cells stain (Thermo Fisher Scientific, USA) was used. Cellular events were measured on a Gallios cytometer and analyzed using Kaluza software (both Beckmann Coulter, USA). Percentage of NK cell subsets and mean fluorescent intensity ratios for NKG2D expression were calculated from CD45^+^ lymphocyte fraction.

### Histology

Paraffin sections of primary HNSCC tumors were stained with anti-human CD3 (clone SP7; Thermo Scientific, USA), anti-human CD8 (clone C8/144B; Dako), anti-human CD20 (clone L26; Dako), anti-human CD68 (clone PG-M1; Dako), anti-human CD56 (clone 123C3; Invitrogen), and anti-human CD163 (clone MRQ-26; Cell Marque) antibodies using the BOND-MAX automated IHC-stainer (Leica Biosystems) according to the manufacturer’s instructions in the Institute of Pathology (University of Cologne, Germany). Numbers of infiltrating cells in the invasive margin and tumor core of HNSCC sections were analyzed separately. Cell counts per high power field (HPF) at 400× magnification were assessed by an experienced pathologist in five HPF of invasive margin and tumor core of every slide, respectively, and the mean positive cell count per HPF was calculated.

### Statistical Analysis

Statistical analyses were performed using Prism 6 software (GraphPad, USA). Data are presented as mean ± SEM as indicated. Statistical significance was evaluated using one-way ANOVA with repeated measurements and *post hoc* test after Bonferroni or unpaired Student’s two-tailed *t*-test as indicated. Differences were considered statistically significant at *p* < 0.05.

## Results

### Elevated sNKG2DL Plasma Levels Correlate with Disease Stage in HNSCC

Previously, we could show a correlation of high sMICA plasma levels and impaired NK cell cytotoxicity in patients with HNSCC (33, 41). Since these studies only investigated sMICA in NKG2D-dependent tumor immune escape, in the current study, we determined the levels of all the shed NKG2DLs (sMICA, sMICB, and sULBP1–3) in plasma samples of 44 HNSCC patients (patients’ characteristics are summarized in Table 1).

**Figure 1:**
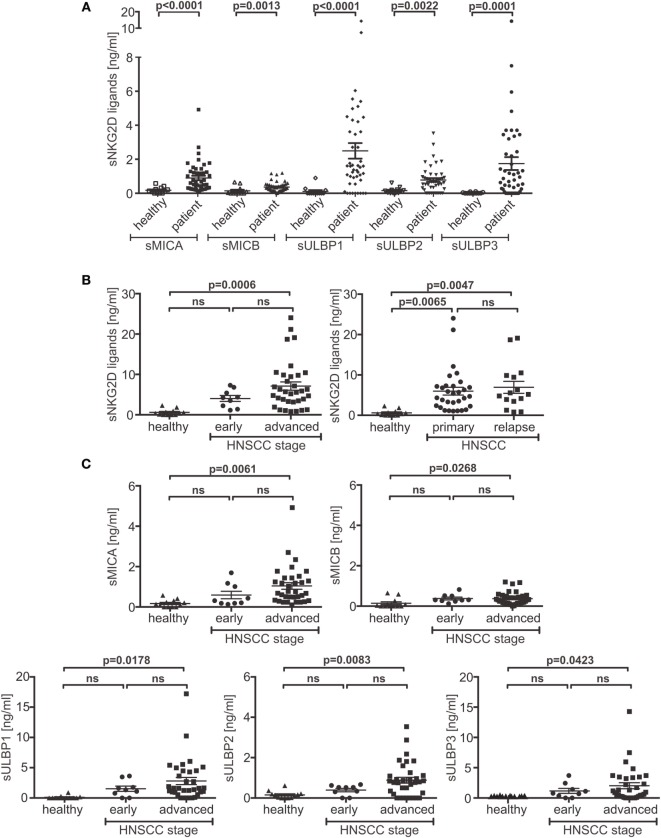
**Disease stage-dependent increase of shed NKG2DL (sNKG2DL) levels**. **(A)** Levels of sNKG2DLs in plasma of head and neck squamous cell carcinoma (HNSCC) patients (*n* = 44) compared to age-matched healthy donors (*n* = 12) quantified by ELISA. **(B)** Cumulative sNKG2DL levels (sum of sMICA/B and sULBP1–3) grouped according to disease stage (left: early = stage I + II; advanced = III + IV) or primary vs. relapsed HNSCC (right). **(C)** Plasma levels of individual sNKG2DLs grouped according to disease stage (early = stage I + II; advanced = III + IV). Data are presented as mean ± SEM of representative experiments. Statistical significance was assessed by unpaired, two-tailed Student’s *t*-test **(A)** and one-way analysis of variance **(B,C)**. ns, non-significant.

Significantly elevated levels of individual sNKG2DLs were detected in patients’ plasma when compared to those observed in the plasma of 12 age-matched healthy donors (Figure 1A). Moreover, the burden of sNKG2DLs in patients with advanced disease vs. healthy donors was significantly increased, whereas no significant difference between primary and relapsed HNSCC was observed (Figure 1B). Comparison of treatment naïve patients with patients currently under palliative chemo- or radiotherapy or after tumor resection showed a therapy induced reduction of sNKG2DL levels (Figure S1A in Supplementary Material). Notably, sNKG2D ligands were found to peak in the plasma of patients with advanced disease (Figure 1C), suggesting that the total level of sNKG2DLs represents an important clinical parameter (Figure S1B in Supplementary Material).

### Immunosuppressive and Proinflammatory Cytokines in HNSCC Patients

**Figure 2:**
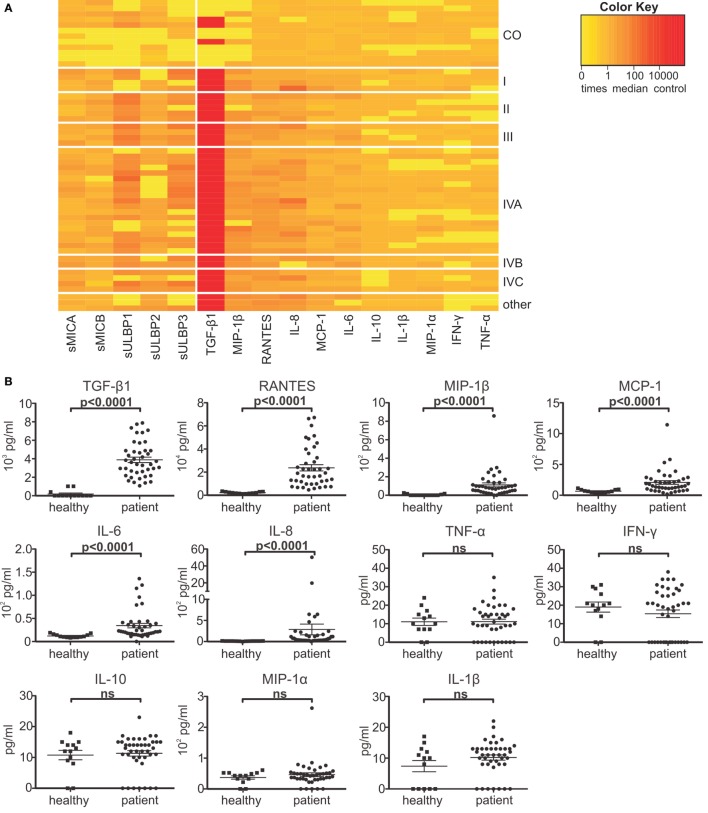

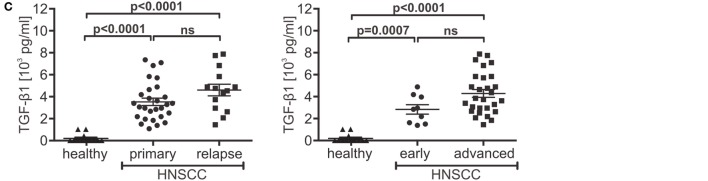
**Elevated immunosuppressive factors in head and neck squamous cell carcinoma (HNSCC) patients**. Patients’ plasma was analyzed for the levels of the cytokines TGF-β1, IL-6, IL-8, IL-10, IL-1β, IFN-γ, TNF-α and the chemokines MIP-1α, MIP-1β, RANTES, and MCP-1 by cytometric bead array. **(A)** Heat-map representation of shed NKG2DL levels (see Figure 1), cytokine and chemokine levels as x-times median control (CO; *n* = 12 healthy donors) grouped according to disease stage (I–IVC). Color code: yellow = normal; orange = elevated; and red = highly elevated. **(B)** Quantification of chemokine and cytokine levels compared to healthy controls. Data are presented as mean ± SEM. **(C)** TGF-β1 plasma levels grouped according to primary vs. relapsed (left) or disease stage (right: early = stage I + II; advanced = III + IV). Statistical significance was assessed by unpaired, two-tailed Student’s *t*-test **(B)** and one-way analysis of variance **(C)**. ns, non-significant.

To further assess the immune status of HNSCC patients, we analyzed the plasma levels of indicative cytokines and chemokines. Elevated plasma levels could be detected for the immunosuppressive and proinflammatory factors TGF-β1, MIP-1β, RANTES, IL-8, MCP-1, and IL-6. However, compared to the plasma levels of healthy controls, HNSCC patients showed no significant differences in the levels of IFN-γ, TNF-α, IL-10, IL-1β, and MIP-1α (Figures 2A,B). Moreover, aberrant cytokine or chemokine levels were not correlated to disease stage (Figure 2A) or primary vs. relapsed HNSCC, except for TGF-β1 showing an increase between stage I and IV (2677 vs. 5358 pg/ml) (Figure 2C).

### Inhibition of NK Cell Cytotoxicity by sNKG2DLs in Patients’ Plasma

**Figure 3:**
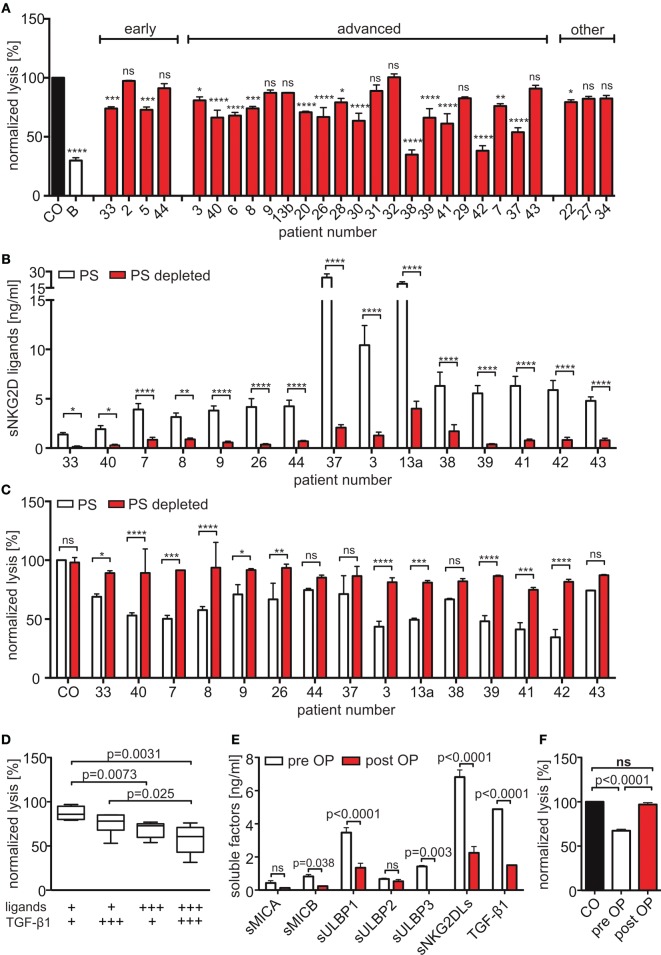
**Shed NKG2DLs (sNKG2DLs)-dependent inhibition and restoration of natural killer (NK) cell cytotoxicity**. **(A)** Cytotoxicity assay of NK cells with CFSE-labeled SiHa target cells (E:T ratio 15:1; 2 h). NK cells were pre-incubated with head and neck squamous cell carcinoma plasma, healthy plasma (CO), or natural killer group 2D (NKG2D) blocking antibodies **(B)**. Cell lysis was calculated by analysis of dead (CFSE^+^/SytoxBlue^+^) and viable (CFSE^+^/SytoxBlue^−^) target cells. Data were normalized to mean of healthy controls and are represented as mean ± SEM of triplicates of a representative experiment. **(B)** The sum of sNKG2DL plasma levels pre-/post-depletion presented as mean ± SEM of duplicates. **(C)** Cytotoxicity assays with NK cells pre-incubated with non-(PS) or NKG2D ligand (NKG2DL)-depleted patients’ plasma (PS depleted) according to **(A)**. **(D)** Lysis of target cells grouped according to sNKG2DLs (+low = 0–2.5 ng/ml and +++ high >2.5 ng/ml) and TGF-β1 (+low = 0–3,000 pg/ml and +++ high >3,000 pg/ml) plasma levels. **(E)** Plasma levels of sNKG2DLs and TGF-β1 of one patient pre-/post-surgery presented as mean ± SEM of duplicates. **(F)** Cytotoxicity assay of NK cells pre-treated with plasma pre-/post-surgery according to **(A)**. Statistical significance was assessed by two-way analysis of variance (ANOVA) **(A–C,E)** or one-way ANOVA **(D,F)**, ns, non-significant, *p = 0.01–0.05, **p = 0.001–0.01, ***p < 0.001, and ****p < 0.0001.

To investigate the inhibitory potency of sNKG2DLs on NK cell cytotoxicity, we performed cytotoxicity assays with primary human NK cells after pre-incubation with plasma from HNSCC patients, healthy controls, or anti-NKG2D blocking antibodies. As target cells, a human cervix carcinoma cell line (SiHa) was used whose killing is strongly dependent on NKG2D. Two-thirds of HNSCC plasma samples significantly diminished target cell killing compared to plasma of healthy donors (Figure 3A). To verify the sNKG2DL-dependency, we depleted sMICA/B and sULBP1-3 from patients’ plasma with antibody-coupled beads. The ligands were removed with efficacies between 61.9 and 93.2% (Figure 3B). Depletion of sNKG2DLs efficiently restored NK cell cytotoxicity (Figure 3C; Figure S2A in Supplementary Material), whereas depletion of healthy plasma had no effect. Thus, NK cell inhibition is mostly due to blocking and downregulation of the NKG2D receptor on NK cells (Figures S2B,C in Supplementary Material). Since we found high levels of TGF-β1, which is known to inhibit NKG2D (42, 43), we correlated the cytotoxic capacity of pre-incubated NK cells with the plasma levels of sNKG2DLs and TGF-β1. High levels of both, sNKG2DLs (>2.5 ng/ml; median: 7.6 ng/ml) and TGF-β1 (>3,000 pg/ml; median: 5,319 pg/ml) showed an additive effect on NK cell inhibition when compared to plasma with low sNKG2DLs (<2.5 ng/ml; median: 1.4 ng/ml) and TGF-β1 (<3,000 pg/ml; median: 2,045 pg/ml) levels (Figure 3D). This is in accordance with our previous results demonstrating an additive effect of sMICA and TGF-β1 on NKG2D-dependent inhibition of NK cell cytotoxicity in HNSCC (33, 41). Nevertheless, the sole depletion of sNKG2DLs was sufficient to restore NK cell cytotoxicity, assuming shedding of ligands as a key mechanism of NKG2D-dependent tumor immune escape in HNSCC. Moreover, plasma samples of one representative patient showed significantly reduced levels of sNKG2DLs and TGF-β1 after surgery (Figure 3E), which were correlated with increased NK cell cytotoxicity (Figure 3F). These findings demonstrate a direct link between tumor burden, NKG2DL shedding and TGF-β1 release and was supported by a patient with recurrent disease (Figures S2D,E in Supplementary Material).

### sNKG2DLs Diminish NK Cell Cytotoxicity and Infiltration of Tumor Spheroids

Next, we analyzed NKG2D-dependent tumor immune escape in tumor spheroids, which resemble many features of poorly vascularized and avascular regions of solid tumors or micrometastases and allow for systematic studies of molecular parameters in a highly controlled microenvironment (39, 44, 45). Tumor spheroids were grown from HNSCC (FaDu, CAL27, and Detroit562) and SiHa (39) cell lines, which show characteristic NKG2DL expression patterns and are highly susceptible to NKG2D-dependent killing (Figures S3 and S4A,B in Supplementary Material).

**Figure 4:**
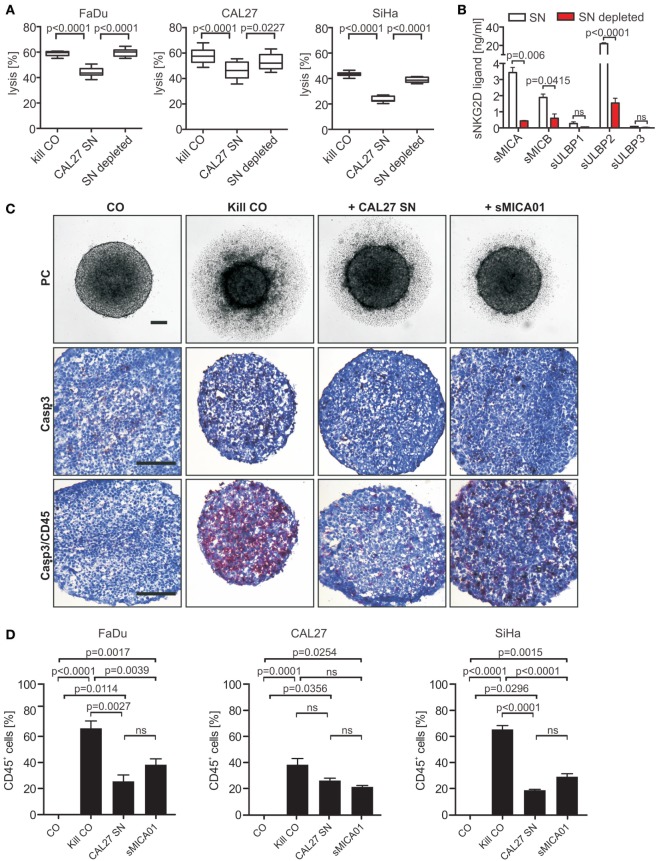
**Natural killer (NK) cell cytotoxicity and infiltration toward tumor spheroids is inhibited by shed NKG2DLs (sNKG2DLs)**. **(A)** NK cells incubated with non-/sNKG2DL-depleted CAL27 supernatant (SN) or medium (kill CO) prior to co-incubation with FaDu, CAL27, or SiHa tumor spheroids (E:T ratio 5:1, 48 h). Tumor spheroid lysis was calculated by analysis of live (CFSE^+^/SytoxBlue^−^) and dead (CFSE^+^/SytoxBlue^+^) tumor cells. Bars correspond to mean ± SEM of eight tumor spheroids. **(B)** sNKG2DL levels of non-/depleted CAL27 SN analyzed by ELISA. Data are shown as mean ± SEM of duplicates. **(C)** NK cells treated with CAL27 SN, sMICA01, or untreated (kill CO) prior to coculturing with FaDu cells for 48 h. Representative phase-contrast pictures at 50× magnification are shown (size bar = 100 µm). Cryosections of spheroids were stained for NK cells (anti-CD45 antibody, red) and apoptosis (anti-active caspase-3 antibody, brown). Representative pictures are shown at 200× magnification (size bar = 200 µm). **(D)** Percentage of infiltrated NK cells in FaDu, CAL27, or SiHa tumor spheroids. Bars correspond to mean ± SEM of 10 vision fields. Statistical significance was assessed by one-way analysis of variance **(A)** and unpaired, two-tailed Student’s *t*-test **(D)**. ns, non-significant.

To investigate the impact of sNKG2DLs on NK cell cytotoxicity, tumor spheroids were cocultured for 48 h at an E:T ratio of 5:1 with NK cells pre-incubated with a cocktail of sNKG2DLs (CAL27 SN: 27.63 ng/ml sNKG2DLs) or sNKG2DLs-depleted CAL27 supernatant (sNKG2DLs: 2.66 ng/ml) (Figures 4A,B). NK cells in basal culture medium served as controls. NK cells efficiently destroyed tumor spheroids (Figure 4A; Figure S4C in Supplementary Material). By contrast, in the presence of high levels of sNKG2DLs, NKG2D-dependent killing was reduced by 25% for FaDu, 19% for CAL27, and 46% for SiHa tumor spheroids (Figure 4A). Importantly, impaired tumor spheroid destruction and NKG2D-downregulation could be completely restored by specific depletion of sNKG2DLs (Figure 4A; Figure S4D in Supplementary Material).

Since antitumor activity of NK cells correlates with infiltration capacity into tumors (46, 47), we investigated NK cell infiltration into tumor spheroids in the absence and presence of high levels of sNKG2DLs. Untreated NK cells (kill CO) or NK cells pre-treated with CAL27 SN or purified sMICA (20 ng/ml) were mixed with HNSCC cells at a 1:1 ratio. Tumor spheroids were monitored by light microscopy for 48 h. In the absence of sNKG2DLs, NK cell killing was accompanied by massive infiltration into tumor spheroids indicated by staining of tumor spheroid cryosections with CD45-specific antibodies (Figure 4C; Figures S5A,B in Supplementary Material). Roughly 66, 38, and 65% of the cells comprising the FaDu, CAL27, or SiHa tumor spheroids, respectively, were NK cells (Figure 4D). In contrast, under inhibitory conditions (in the presence of sMICA or CAL27 SN), NK cell cytotoxicity, shown as tumor spheroid volume reduction (Figure S5C in Supplementary Material), was inhibited. This inhibition was accompanied by drastically reduced NK cell infiltration with only 26, 26, or 19% of the cells of FaDu, CAL27, or SiHa tumor spheroids being NK cells, respectively (CAL27 SN; Figure 4D). Interestingly, the same amount of sMICA alone was sufficient for NK cell inhibition, with 38, 21, and 29% of NK cell infiltration for FaDu, CAL27, and SiHa tumor spheroids, respectively, when compared to the cocktail of shed ligands in the CAL27 SN, suggesting that the total burden of NKG2DLs might be crucial for NK cell dysfunction in cancer patients. Additionally, infiltrated pre-treated NK cells were often double positive for CD45 and cleaved caspase-3 compared to untreated NK cells. Therefore, we speculate that under inhibitory conditions NK cells might proliferate less and become apoptotic. Moreover, both NK cell subtypes (CD56^bright^/CD16^+/−^; CD56^dim^/CD16^+^) could infiltrate into tumor spheroids (Figure S5D in Supplementary Material).

### Low NK Cell Infiltration of Primary HNSCC

**Figure 5:**
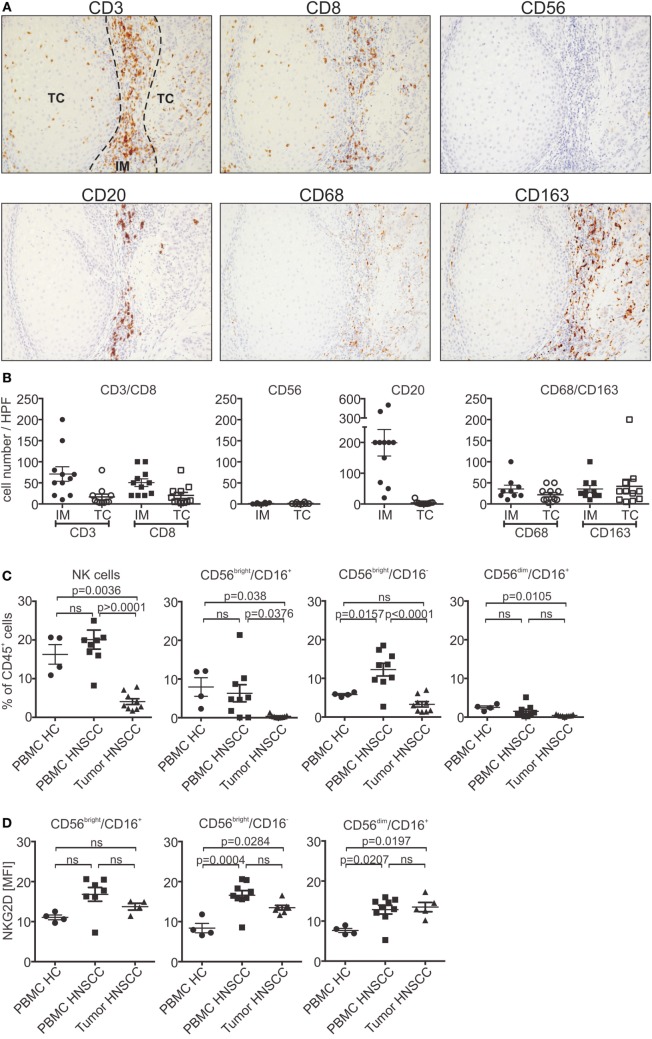
**Low infiltration of lymphocytes in primary head and neck squamous cell carcinoma (HNSCC)**. **(A)** Paraffin sections of primary HNSCC tumors stained for CD3^+^/CD8^+^, CD20^+^ or CD56^+^ lymphocytes, or CD68^+^/CD163^+^ macrophages. Consecutive sections of a representative patient are shown (magnification 200×). **(B)** Number of infiltrating lymphocytes and macrophages quantified by counting infiltrating cells in High power fields of the tumor slides. Total cell numbers/HPF are plotted as mean ± SEM (*n* = 11 patients). **(C)** Flow cytometric analysis of natural killer (NK) cell subpopulations (CD56^bright^/CD16^+/−^ and CD56^dim^/CD16^+^) of patients’ peripheral blood mononuclear cells (PBMCs) and corresponding tumor tissues. PBMCs from healthy donors served as control. NK cell numbers were calculated and plotted as % of CD45^+^ lymphocytes (*n* = 9 patients; *n* = 4 healthy controls). **(D)** Corresponding natural killer group 2D (NKG2D) expression levels of NK cell subpopulations plotted as mean fluorescent intensity (MFI) over background (*n* = 9 patients). Statistical significance was assessed by one-way analysis of variance. ns, non-significant.

To further investigate tumor infiltration *in vivo*, primary HNSCC tumor sections were stained for CD3^+^ and CD8^+^ T cells, CD20^+^ B cells and CD56^+^ NK cells, as well as CD68^+^/CD163^+^ macrophages (Figures 5A,B). Infiltrating cells were analyzed separately for the invasive margin (IM) or tumor core (TC). T and B cells were predominantly found in the IM (CD3 T cells: mean 71 cells/HPF; CD8 T cells: mean 50 cells/HPF; CD20 B cells: 200 cells/HPF). In contrast, only a small proportion of lymphocytes could infiltrate into the tumor core (CD3 T cells: mean 17 cells/HPF; CD8 T cells: mean 20 cells/HPF; CD20 B cells: 4 cells/HPF). No differences could be observed for CD8^+^ T cells when compared to the whole CD3^+^ T cell population. Moreover, tumor-associated macrophages were equally distributed throughout the tissue. Interestingly, only very low numbers or a complete absence of NK cells could be found in the tumor tissues (IM/TC: mean 2 cells/HPF). The low infiltration potential of the different lymphocyte populations is indicative for the insufficient tumor rejection in HNSCC patients. For further analysis, NK cells of HNSCC patients’ PBMCs and corresponding dissociated tumor samples were subjected to flow cytometry experiments. The total number of circulating NK cells in the CD45^+^ lymphocyte population of PBMCs was nearly unchanged compared to healthy controls (Figure 5C). Regarding regulatory (CD56^bright^/CD16^+/−^) and cytotoxic (CD56^dim^/CD16^+^) NK cells, HNSCC patients showed a slight shift toward the CD56^bright^ NK cell subpopulation. Analysis of tumor samples confirmed the histological staining showing that only a minor proportion of tumor-associated lymphocytes were NK cells. Interestingly, the infiltrated NK cells were predominantly from the regulatory subpopulation, whereas cytotoxic NK cells were nearly absent. Moreover, analysis of NKG2D expression levels showed a small increase compared to healthy controls of maximum twofold for the CD56^bright^ population (Figure 5D).

### Preclinical Evaluation of Adsorption Apheresis of sNKG2DLs in Rhesus Macaques

**Figure 6:**
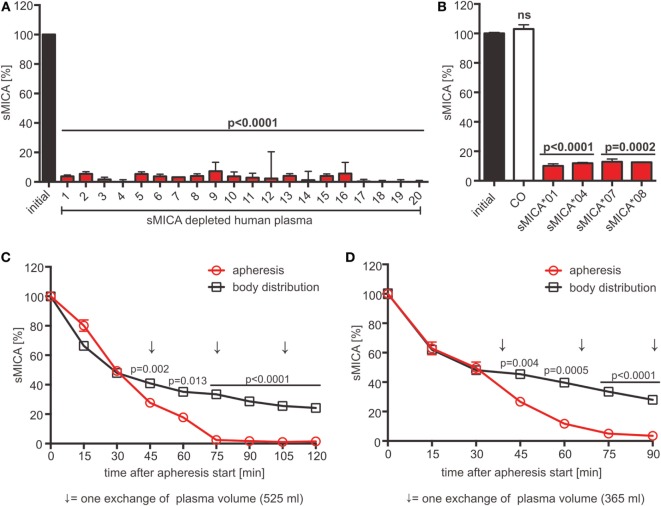
**sMICA depletion by adsorption apheresis using an anti-MICA antibody**. **(A)** Depletion of sMICA*04 (20 ng/ml) from human plasma (*n* = 20 donors) with antibody-coupled beads. sMICA*04 plasma levels post-depletion measured by ELISA and normalized to initial sMICA*04 level. Data represented as mean ± SEM of duplicates of a representative experiment. **(B)** Depletion of sMICA alleles (sMICA*01, sMICA*04, sMICA*07, and sMICA*08) from C1R supernatants. Beads with mouse IgG_1_ antibody served as control (CO). sMICA levels measured by ELISA and normalized to initial sMICA. Data represented as mean ± SEM of duplicates of a representative experiment. **(C,D)** Animals were injected with 100 µg/l blood volume sMICA*04. Apheresis was started 15 min post-injection using a Life18™ apheresis unit (Miltenyi Biotec) equipped with an adsorber cartridge (anti-MICA antibody covalently coupled to sepharose). sMICA plasma concentrations were determined by ELISA every 15 min and normalized to initial sMICA (15 min post-injection). Data are represented as mean ± SEM of duplicates. Body distribution (pilot study): open square, black; apheresis: open circles, red. Arrows indicate the time point of one plasma volume (PV) exchange [**(C)** PV = 525 ml; **(D)** PV = 365 ml]. Statistical significance was assessed by unpaired, two-tailed Student’s *t*-test.

Our data suggest that the cumulative sNKG2DL level is indicative for the extent of NKG2D-dependent tumor immune escape. Consequently, sNKG2DL plasma depletion by adsorption apheresis might be a clinical intervention strategy to significantly boost antitumor efficacy of autologous and, if combined with cell-based therapies, adoptively transferred or allogeneic immune cells. For proof-of-concept studies, we employed a human sMICA-specific antibody, raised by immunizing BALB/c mice with a mixture of MICA*01 and MICA*04 (38), with an epitope in the α1-helical domain (Figures S6A,B in Supplementary Material). To test its depletion efficacy, human plasma of 20 healthy donors supplemented with recombinant sMICA*04 (production and purification of sMICA*04, see Figures S6C–E in Supplementary Material) or cell culture SN containing allelic sMICA variants were incubated with antibody-coupled magnetic beads. The antibody showed a depletion efficacy of 89–97% of sMICA allelic variants frequently found in the Caucasian population (48) independent of the donor immune status (Figures 6A,B). Moreover, beside sNKG2DLs also exosomes containing NKG2DLs (MICA*08, Figure 6B) (49) could be efficiently removed.

For preclinical validation of adsorption apheresis, we used a rhesus macaque (*Macaca mulatta*) model. Human and rhesus MICA display a sequence identity of 79%, human and rhesus NKG2D a sequence identity of 94% (Figures S6F,G in Supplementary Material). In a pilot study, two rhesus monkeys were injected with 100 µg sMICA*04 per liter blood volume, and sMICA plasma levels were analyzed over 3 h. About 15–25% of the initially injected sMICA could be found in the plasma 15 min after injection. An exponential decrease of sMICA could be observed during the first 150 min reaching a stable plasma level with diffusion equilibrium of about 3–5 ng/ml after 3 h, which resembles the plasma levels found in cancer patients (Figure S7A in Supplementary Material). Notably, no renal clearance, proteolytic degradation of sMICA (Figures S7B,C in Supplementary Material), or immune reaction against sMICA was observed (Figure S7D in Supplementary Material). These data indicate that the decreasing sMICA plasma levels reflect equilibrium formation of sMICA’s body distribution.

For adsorption apheresis, two animals were injected with sMICA*04, and the apheresis was started 15 min post-injection until three exchanges of the animals’ plasma volume were reached. Analysis of sMICA plasma levels showed a nearly complete depletion of sMICA from the plasma after only three plasma volume exchanges with 1.44 ± 0.06 and 3.48 ± 1.79% remaining sMICA*04 when compared to the endpoints reached by body distribution without treatment (24.17 ± 0.09 and 27.98 ± 0.46% remaining sMICA*04 in the two animals, respectively) (Figures 6C,D). Notably, since the two monkeys were healthy without signs of immune challenge at the time of the experiment, the basal level of NKG2D on the monkeys’ T and NK cells was not affected by human sMICA (Figures S7E–G in Supplementary Material). In conclusion, we suggest that adsorption apheresis of sNKG2DLs from patients’ plasma might be a promising combinatorial medical approach to overcome NKG2D-dependent tumor immune escape and to improve the efficacy of (cellular) cancer immunotherapy.

## Discussion

Natural killer group 2D-dependent tumor immune escape mechanisms contribute to impaired tumor surveillance. Previously, we could show a correlation of increased sMICA and TGF-β1 plasma levels with diminished NK cell cytotoxicity in untreated HNSCC patients (33, 41). NKG2D-dependent immune escape based on the release of sNKG2DLs has been described in a variety of cancers (50, 51), but most studies mainly concentrated on sMICA and sMICB. Therefore, the knowledge of the cumulative sNKG2DL levels in cancer patients and their impact is still scarce. In the present study, profiling of HNSCC plasma revealed that the cumulative level of the sNKG2DLs (sMICA/B and sULBP1-3) is patient specific, correlates with disease progression and tumor load, and is indicative for NKG2D-dependent tumor immune escape in HNSCC. This is in accordance with studies showing that high sMICA and sMICB plasma levels are associated with tumor progression and poor prognosis in advanced oral squamous cell carcinoma (32, 52) and other malignancies such as prostate cancer and multiple myeloma (31, 53). The accumulation of sNKG2DLs in advanced diseases might be associated with the upregulation of matrix metalloproteinases, which mediate proteolytic cleavage of NKG2DLs, with increased tumor invasiveness (54). Moreover, our data suggest a prominent role for sULBP1 and sULBP3 in HNSCC when compared to sMICA/B. The diverse NKG2DL expression patterns in different cancer entities and in individual patients might be explained by differential transcriptional regulation, which can be influenced by many factors of the tumor microenvironment and cancer therapies (55–57). Interestingly, palliative chemo- and radiotherapy had little effect on sNKG2DL levels. In addition, altered cytokine and chemokine levels in HNSCC patients were highly heterogeneous with only TGF-β1 being disease stage dependent, but commonly showed an immunosuppressive, proinflammatory, and proangiogenic phenotype (58, 59). Moreover, high numbers of tumor-associated macrophages, predominantly of the M2 phenotype, found in primary HNSCC tumor tissues further corroborated the immunosuppressive state of HNSCC. In contrast to previous reports (33, 58, 60, 61), we could not detect significant alterations in the levels of TNF-α, IL-10, or IFN-γ. It should be noted that analysis of plasma from a relatively small patient cohort with different tumor localization, stages, additional carcinogens (e.g., HPV-infection, smoking) and treatments might contribute to the divergent results. Lathers and Young (58) showed in a study analyzing HNSCC plasma of 101 patients a bias toward Th2 cytokines (e.g., IL-4, IL-6, and IL-10), but a loss of interrelationship between Th1/Th2 cytokines toward advanced disease when compared to healthy controls. The shift toward Th2 cytokines is in accordance with other studies (61, 62) and our results of patients’ plasma and primary tumor sections. Moreover, Green et al. (62) could show that HNSCC treatment reduced Th2 cytokines in the plasma of 101 HNSCC patients, while not increasing Th1 cytokines confirming a rebalance of Th1/Th2 cytokines in HNSCC. However, the results of the different studies on cytokine levels in HNSCC varied markedly for individual factors underlining the heterogeneous nature of this cancer. The results on cytokine levels could vary using different assays and depends on the material analyzed, since the complex cytokine and chemokine network is modulated by different cell types such as tumor cells, stromal cells, and immune cells and depends on the cellular cross-talk of these cells (59). In this respect, Pries and colleagues (63) could show that HNSCC tumor cell lines alone could secrete high levels of IL-6/IL-8 but no significant levels of IL-4 and IL-10 in the absence of infiltrating immune cells. Moreover, Bose et al. (61) showed cytokines analyzed from mononuclear cell culture supernatants isolated from HSNCC patients rather than from HNSCC plasma samples. Therefore, comparison of different studies might be difficult and a uniform diagnostic method for clinical use should be established to further evaluate the immune status in a large patient cohort.

Previous studies showed a NKG2D-dependent NK and T cell inhibition through sMICA/B molecules in different cancer entities (24, 28, 33, 50, 64). In our study, HNSCC patients’ plasma led to reduced NK cell cytotoxicity by blocking the NKG2D receptor. We could see an additional inhibitory effect when HNSCC plasma contained high levels of both, sNKG2DLs and TGF-β1 [current study and in Ref. (33)]. This is in accordance with studies showing that suppression of NKG2D-mediated antitumor functions can be correlated with high TGF-β1 plasma levels in neuroblastoma and colorectal cancer patients (42, 43). Moreover, tumor resection in one patient led to reduced sNKG2DL and TGF-β1 plasma levels and increased NK cell cytotoxicity. These results are supported by a study of Crane et al. showing restored NKG2D-dependent cytotoxicity of NK and T cells after tumor resection in glioma patients (65). Importantly, we could show that specific depletion of sNKG2DLs could completely restore NKG2D-dependent killing of monolayer cell cultures and 3D tumor spheroids, demonstrating that NK cell dysfunction is predominantly governed by shed NKG2DLs. Interestingly in case of patient 37, high sNKG2DLs and a median TGF-β1 level showed only moderate NK cell inhibition (prior to ligand depletion) and depletion of sNKG2DLs from plasma had only a minor effect on restoration of NK cell cytotoxicity. A marginal increase in NK cell cytotoxicity after sNKG2DL depletion, which was not significant, could also be observed in three more samples. It is interesting that in these cases NK cells showed relatively high cytotoxicity toward tumor cells before sNKG2DL depletion compared with NK cells incubated with healthy plasma. In this respect, it was shown that NKG2D expression can be increased by different cytokines including IL-2, IL-12, IL-15, and type I interferons (66). Moreover, it cannot be excluded that these plasma samples contain factors which act on other inhibitory and/or activating NK cell receptors and, therefore, partially counteract the effect of sNKG2DLs and/or TGF-β1. However, our further analyses of cytokine and chemokine plasma levels could not give any additional hints on these factors. Regarding the complexity of human plasma and the complexity of the NK cell receptor system, which regulates NK cell cytotoxicity, the identification of all components regulating NK cell functions is hard to address and needs further investigations. On the other hand, the composition and nature of sNKG2DL levels might play a role for the extend of NK cell inhibition. In this respect, it could be shown that NKG2DLs released on exosomes triggered significantly more NKG2D-donwregulation than monovalent sNKG2DLs (49, 67, 68). In accordance with that, Clayton et al. could show that NKG2DLs and TGF-β1 released from mesothelioma cell-generated exosomes markedly decreased NKG2D surface expression on activated NK and T cells (69). Notably, the inhibitory impact of TGF-β1 on NK cells *in vivo* might be stronger by acting through the suppressive function of Tregs (70), which are increased in HNSCC patients as shown by Bose et al. (61). Therefore, profiling of sNKG2DLs and TGF-β1 as diagnostic/prognostic markers might be relevant for individualized therapy to decipher the time point and patient cohort to benefit from an intervention strategy for NKG2D-dependent tumor immune escape.

Using tumor spheroids (39), we could show for the first time a correlation between NKG2D-dependent NK cell inhibition and decreased infiltration. Interestingly, the same amount of shed MICA, purified from supernatant of tumor cells, inhibited NK cell cytotoxicity and infiltration to the same extent as a cocktail of sNKG2DLs. This supports the hypothesis that the composition of sNKG2DLs and especially the level of high-affinity ligands in the plasma might be important for the extend of NK cell inhibition. However, the detailed mechanism of sNKG2DL-dependent suppression of NK cell infiltration needs further investigation. One possible mechanism could be NK cell exhaustion through NKG2D-downregulation resulting in low NK cell functions and viability as reported by Rossi et al. showing a correlation of NKG2D and NKp46 downregulation and decreased NK cell viability and function after histone deacetylase inhibitor treatment (71). The reduced infiltration into tumor spheroids also reflects the situation in primary tumors of HNSCC patients. Whereas low numbers of CD3^+^/CD8^+^ and CD20^+^ tumor-infiltrating lymphocytes could be found, nearly no infiltration of CD56^+^ NK cells (and presumably NKT cells) could be detected. This is in accordance with a study showing low NK cell infiltration in primary tumor tissues and regional lymph nodes in oral cancer patients (72). Moreover, HNSCC patients had decreased numbers of peripheral cytotoxic CD56^dim^/CD16^+^ NK cells and a shift toward CD56^bright^ NK cells. A bias toward CD56^bright^ NK cell subpopulation and reduced CD16 expression was also described for patients with advanced cancers, such as melanoma, breast cancer, esophageal squamous cell carcinoma, and pediatric leukemia (73–76). The reduction in CD16^+^ NK cell subsets is further correlated to decreased NK cell cytotoxicity and the immunosuppressive milieu of advanced cancers (73, 74, 77). Tumor infiltration of NK cells is associated with a better prognosis in several cancer entities, such as colorectal cancer, non-small cell lung cancer, and clear cell renal cell carcinoma (78–82). For HNSCC, several studies showed a positive correlation of high NK cell infiltration, especially in HPV^+^ HNSCC, in primary tumors, and overall survival rates (83–85). Thus, low numbers of infiltrated NK cells might be one explanation for insufficient HNSCC immunosurveillance, and these patients might benefit from sNKG2DL depletion.

In our *in vitro* cytotoxicity assays, we could show that depletion of sNKG2DLs efficiently restored NK cell functions. In a proof-of-concept study with rhesus monkeys, we have shown that quantitative removal of sMICA can be achieved by adsorption apheresis. Therefore, we suggest adsorption apheresis of sNKG2DLs as a supportive therapy for HNSCC patients in order to restore cytotoxicity of autologous NKG2D^+^ immune cells, such as NK cells, NKT cells, and T cells. Moreover, adsorption apheresis prior to administration of cellular therapies might help to maintain and prolong the cytotoxic activity of adoptively transferred and allogeneic immune cells after haploidentical stem cell transplantation, which can be affected by sNKG2DLs (28), and favor the success of immune cell therapies. Besides healthy donor derived cell products this will also include CAR-functionalized T cells and NK cells.

Currently, several lines of argumentation indicate that adsorption apheresis of sNKG2DLs might be a powerful supportive intervention in cancer therapy. First, one individual patient post surgery had significantly lower sNKG2DL plasma levels than before surgery (Figure 3E). This demonstrates that the overall sNKG2DL level correlates with tumor burden and that tumor load determines the maximum level of the equilibrium of plasma sNKG2DLs. Second, after apheresis, the fraction of NKG2D^+^ immune cells that are capable of attacking NKG2DL^+^ tumor cells will be drastically increased. In turn, the tumor mass and its capacity to reproduce sNKG2DLs will be reduced. Third, since adsorption apheresis represents a minimal invasive procedure similar to hemodialysis of diabetic patients, we assume that it can be repeated several times after cellular therapy in order to scavenge reappearing sNKG2DLs from patients’ plasma. Previous results indicate that sMICA plasma levels in pediatric neuroblastoma patients’ plasma remained low for more than 72 h after adoptive transfer of NK cells from healthy donors most likely due to scavenging of sMICA on NKG2D in the plasma membrane of the transferred NK cells (28). Therefore, we expect that periodic “refreshing” of the NKG2D^+^ immune cells will lead to constant shrinking of the tumor mass, sustained reduction of sNKG2DL plasma levels, and extended antitumor activity of NKG2D^+^ immune cells.

## Ethics Statement

Plasma of patients and healthy volunteers was analyzed after written informed consent from all subjects and in accordance with and approved by the Ethics Committee of the University Clinic Cologne (Immucan 11-116, 2011). Animal experiments were carried out in accordance with and with approval by the Niedersächsisches Landesamt für Verbraucherschutz und Lebensmittelsicherheit.

## Author Contributions

SW, SM, AL, AG, AQ, and RS performed experiments, SW, JK, TB, AM-R, KL, and LW participated in animal experiments. JK designed the study. SW, AL, AG, RS, EH, MB-B, and JK analyzed the data. SW, MB-B, and JK wrote the manuscript. VH, DB, UK, LW, and AS contributed to manuscript preparation and discussion. All authors have read and approved the final manuscript.

## Conflict of Interest Statement

The authors declare that the research was conducted in the absence of any commercial or financial relationships that could be construed as a potential conflict of interest.
