# Supplementary material for: Natural Killer Group 2D Ligand Depletion Reconstitutes Natural Killer Cell Immunosurveillance of Head and Neck Squamous Cell Carcinoma
Source: Front Immunol. 2017 Apr 10;8:387. doi: 10.3389/fimmu.2017.00387 (PMC5385630; doi:10.3389/fimmu.2017.00387)
Supplement: Supplementary file 1 [file data_sheet_1.pdf]

Supplementary Material

Natural Killer Group 2D Ligand Depletion Reconstitutes Natural Killer Cell Immunosurveillance of Head and Neck Squamous Cell Carcinoma

Sandra Weil[1,2†], Stefanie Memmer[1,2†], Axel Lechner[3], Volker Huppert[4], Ariane Giannattasio[1], Tamara Becker[5], Andreas Müller-Runte[4], Karen Lampe[6], Dirk Beutner[3], Alexander Quaas[7], Ralf Schubert[8], Eva Herrmann[9], Alexander Steinle[10], Ulrike Koehl[11], Lutz Walter[12], Michael S. von Bergwelt-Baildon[13] and Joachim Koch[1,2†*]

[*]Correspondance:

Prof. Dr. Joachim Koch, joachim.koch@uni-mainz.de

Supplementary Materials and Methods

Multicellular Tumor Spheroids

Solid tumor spheroids were generated as described previously (1) by seeding $5x10^3$-$1x10^4$ cells/well in a volume of 150 µl/well of culture medium in 96-well plates coated with 1.5% agarose in basal medium. Solid tumor spheroids formed within approximately 24-48 h after initial seeding (d0) and were used in functional assays. For tumor spheroid growth curves, phase contrast pictures of independent solid spheroids were analyzed for each condition from six independent experiments by Fiji software (2).

Tumor Spheroid Infiltration of NK Cell Subpopulations

For infiltration studies with NK cell subpopulations, primary human NK cells on d7 of culture were stained with anti-human CD56 (clone B159; BD Bioscience, USA) and sorted on a FACS ARIAII instrument (BD Bioscience, USA) for $CD56^{bright}$ and $CD56^{dim}$ subpopulations. Solid FaDu tumor spheroids were co-cultured at an E:T of 1:1 with the individual NK cell subpopulations for 48 h. For histology, individual spheroids (n > 8) were collected and embedded into OCT compound (Sakura, Japan). Cryosections were stained with mouse anti-human CD45 (clone 2B11+PD7/26; DAKO, Germany) and analyzed by standard two-step polymer methods with Envision kits (DAKO, Germany). For counterstaining, Hematoxylin (DAKO, Germany) was used according to manufacturer's instructions.

Cytotoxicity Assays with NKG2D Blocking Antibodies

For blocking experiments, NK cells were pre-incubated with mouse anti-human NKG2D antibodies (MAB139; R&D Systems, USA) for 30 minutes prior to co-incubation with CFSE-labeled HNSCC (co-incubation for 16 h) and SiHa (co-incubation for 2 h) cell lines at appropriate effector-to-target (E:T) ratios in NK cell medium without IL-2. NK cells were labeled with mouse anti-human CD45-APC antibodies (clone 5B1; Miltenyi Biotec, Germany) and live/dead cell discrimination was achieved by staining with SytoxBlue (Life Technologies, USA) prior to the measurement on a BD FACSCanto II instrument equipped with a 96-well plate HTS Sampler. Cellular event counts were analyzed with FlowJo software. The percentage of cell lysis was calculated by evaluation of viable ($CFSE^+$/$SytoxBlue^-$) and dead ($CFSE^+$/$SytoxBlue^+$) target cells. Data is represented as mean ± SEM of triplicates. Statistical significance was determined by one-way ANOVA.

Flow Cytometry of Tumor Spheroids

For detection of NKG2DLs, TrypLE™ Express-treated (Life Technologies, USA) tumor spheroids were stained with mouse monoclonal antibodies: mouse anti-human MICA (clone AMO1) (3)), mouse anti-human MICB (MAB1599), mouse anti-human ULBP1 (MAB1380), mouse anti-human ULBP2 (MAB1298) and mouse anti-human ULBP3 (MAB1517; all R&D Systems, USA). Rat anti-mouse IgG_1-APC (130-095-902; Miltenyi Biotec, Germany) or anti-mouse $IgG_{2a/b}$-APC (130-095-880; Miltenyi Biotec, Germany) served as secondary antibodies. As negative control, samples incubated with secondary antibodies only were used. Cells were measured on a FACSCanto II instrument equipped with a 96-well plate HTS Sampler (BD Bioscience, USA) and analyzed with FlowJo software (Tree Star, USA). Viability of cells was determined by SytoxBlue (Life Technologies, USA) stain. 10.000 events of viable cells were analyzed from individual experiments (n=3) to calculate x-fold MFI over background and displayed as mean ± SEM.

Rhesus monkey lymphocytes were stained from whole blood samples by incubation with a cocktail of conjugated mouse anti-human CD3-APC.Cy7 (clone HIT3a), anti-human CD20-FITC (clone 2H7; both BioLegend, USA) and anti-human CD314-APC (clone BAT221; Miltenyi Biotec, Germany) following erythrocyte lysis using ACK lysis buffer (Thermo Fisher Scientific, USA) according to manufacturer's instructions. Cells were measured on a LSRII instrument (BD Bioscience, USA) and analyzed with FlowJo software to calculate the MFI over background.

Protein Expression and Purification

To generate shed MICA (sMICA01), a DNA fragment encoding full length MICA engineered with N-terminal hexa histidine-tag was cloned into the tet-on vector pES.1-T6-PGKPuro (kindly provided by Dr. Manuel Grez, Georg-Speyer-Haus, Frankfurt am Main, Germany). UKF-NB3 (human neuroblastoma) cells kindly provided by Dr. Ulrike Koehl (MHH, Hannover, Germany) were maintained in IMDM medium (Life Technologies, USA) supplemented with 10% FCS (PAA and PAN Biotech, Germany) and 1% Penicillin/Streptomycin (Life Technologies, USA). UKF-NB3 cells were co-transfected with the plasmids pES.1-T6-PGKPuro-MICA01 and pES1-2(M2N) (M2, rtTA) using the Neon transfection system (Life Technologies, USA). Cells were selected with 0.25 mg/ml G418 and 1 µg/ml puromycin (Life Technologies, USA). MICA expression was induced by addition of 2 µg/ml doxycycline (Sigma, Germany). Cells were stressed by serum starvation for 72 h to induce MICA shedding. sMICA was purified with Protino Ni-NTA agarose

(Macherey-Nagel, Germany) from dialyzed cell culture supernatant and concentrated using Amicon centrifugal filter units with a molecular weight cut-off of 10 kDa.

To generate soluble MICA (sMICA*04), the DNA sequence encoding the MICA*04 ectodomain (aa 23-299) engineered with a C-terminal hexahistidin-tag was cloned into the pFUSE-hIgG1-Fc2 vector (InvivoGen, USA) containing an IL-2 secretion sequence. HEK293T/17 cells (CRL-11268, human embryonic kidney cells) were purchased from the American Type Culture Collection (ATCC) and maintained in DMEM medium (Life Technologies, USA) supplemented with 10% FCS (PAA and PAN Biotech, Germany), 1% Penicillin/Streptomycin (Life Technologies, USA), 2 mM L-Glutamine (Life Technologies, USA). HEK293T/17 cells were transiently transfected using the polyethyleneimine method (4). Secreted sMICA*04 was purified with Protino Ni-NTA agarose and subsequent size exclusion chromatography (SEC) on a HiLoad 16/60 Superdex200 column (GE Healthcare, UK) in PBS using an ÄKTA FPLC system (GE Healthcare, UK). Elution fractions containing the sMICA*04 protein were concentrated using Amicon centrifugal filter units with a molecular weight cut-off of 10 kDa.

Deglycosylation Assay

To check for glycosylation, sMICA*04 was treated with either peptide-N-glycosidase F (PNGaseF) to remove N-linked glycans, or protein deglycosylation mix (both New England Biolabs, USA) for removal of N- and O-linked oligosaccharides according to the manufacturer’s protocol. Deglycosylation was analyzed by molecular mass reduction via SDS-PAGE and immunoblot with mouse anti-MICA antibody (MAB1300; R&D Systems, USA) and anti-mouse IgG HRP-conjugated secondary antibody (Sigma-Aldrich, USA). Recombinant MICA*04 produced from *E.coli* as previously described (5) served as non-glycosylated control.

Peptide Spot Arrays

Peptide spot arrays of human MICA (Q29983 aa23-308; 18 amino acid peptides; off-set by 4 amino acids) were synthesized by Fmoc chemistry at activated PEG spacers on cellulose membranes by automated parallel peptide synthesis on a MultiPep RS instrument (Intavis, Germany) and used for binding experiments as described previously (6, 7). After blocking with 5% milk/TBS-T, peptide membranes were incubated with anti-MICA antibody (clone AMO1). Peptide membranes were analyzed by chemiluminescence imaging after incubation with anti-mouse IgG HRP-conjugated antibody (Sigma-Aldrich, USA).

Rhesus Antibody Detection

For the analysis of anti-MICA monkey IgG/IgM antibodies the animals’ plasma were analyzed three weeks after sMICA*04 injection. Therefore, ELISA plates were coated with sMICA*04, blocked with BSA blocking solution (Candor, Germany) and probed with rhesus macaque plasma in different dilutions and subsequent washing steps with 0.05% Tween20-PBS. Bound antibodies were detected with either anti-monkey-IgG (SAB3700766) or anti-monkey-IgM (SAB3700780) HRP-conjugated antibodies (both Sigma-Aldrich, USA). Following visualization with TMB substrate (KPL, Germany), signals were measured in a microtiter plate reader ($\lambda = 450$ nm) and analyzed with Prism 6 software (GraphPad, USA). Data of plasma samples were calculated as mean ± SEM.

Supplementary References

1. Giannattasio A, Weil S, Kloess S, Ansari N, Stelzer EH, Cerwenka A, et al. Cytotoxicity and infiltration of human NK cells in in vivo-like tumor spheroids. *BMC cancer* (2015) **15**(1):351. doi: 10.1186/s12885-015-1321-y. PubMed PMID: 25933805; PubMed Central PMCID: PMC4422268.

2. Schindelin J, Arganda-Carreras I, Frise E, Kaynig V, Longair M, Pietzsch T, et al. Fiji: an open-source platform for biological-image analysis. *Nature methods* (2012) **9**(7):676-82. doi: 10.1038/nmeth.2019. PubMed PMID: 22743772; PubMed Central PMCID: PMC3855844.

3. Salih HR, Rammensee HG, Steinle A. Cutting edge: down-regulation of MICA on human tumors by proteolytic shedding. *J Immunol* (2002) **169**(8):4098-102. Epub 2002/10/09. PubMed PMID: 12370336.

4. Anliker B, Abel T, Kneissl S, Hlavaty J, Caputi A, Brynza J, et al. Specific gene transfer to neurons, endothelial cells and hematopoietic progenitors with lentiviral vectors. *Nature methods* (2010) **7**(11):929-35. doi: 10.1038/nmeth.1514. PubMed PMID: 20935652.

5. Steinle A, Li P, Morris DL, Groh V, Lanier LL, Strong RK, et al. Interactions of human NKG2D with its ligands MICA, MICB, and homologs of the mouse RAE-1 protein family. *Immunogenetics* (2001) **53**(4):279-87. PubMed PMID: 11491531.

6. Hilpert K, Winkler DF, Hancock RE. Peptide arrays on cellulose support: SPOT synthesis, a time and cost efficient method for synthesis of large numbers of peptides in a parallel and addressable fashion. *Nat Protoc* (2007) **2**(6):1333-49. doi: 10.1038/nprot.2007.160. PubMed PMID: 17545971.

7. Plewnia G, Schulze K, Hunte C, Tampe R, Koch J. Modulation of the antigenic peptide transporter TAP by recombinant antibodies binding to the last five residues of TAP1. *J Mol Biol* (2007) **369**(1):95-107. doi: 10.1016/j.jmb.2007.02.102. PubMed PMID: 17418234. Anliker, B., Abel, T., Kneissl, S., Hlavaty, J., Caputi, A., Brynza, J., et al. (2010). Specific gene transfer to neurons, endothelial cells and hematopoietic progenitors with lentiviral vectors. *Nat Methods* 7(11)**,** 929-935. doi: 10.1038/nmeth.1514.

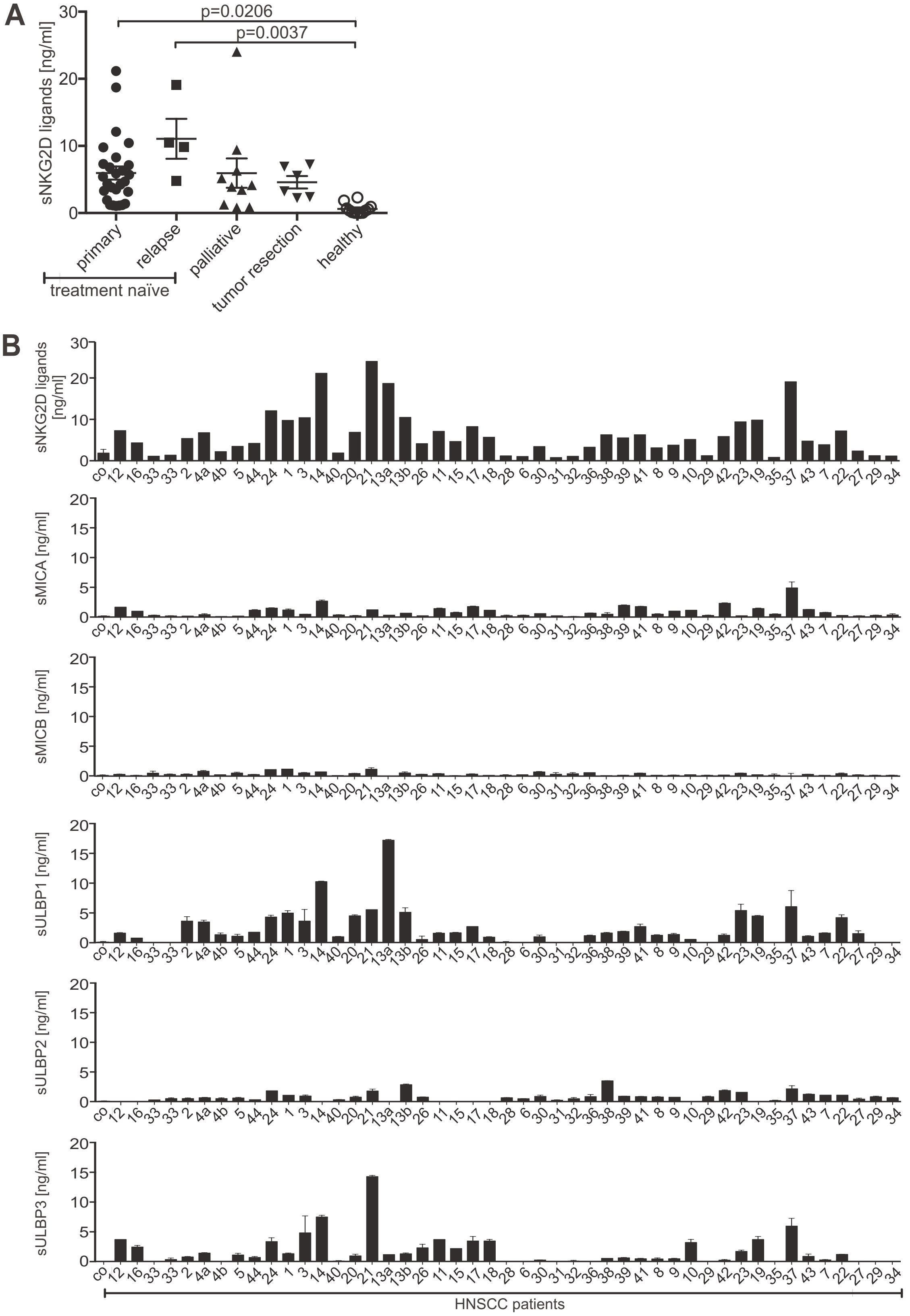

Supplementary Figure S1

Supplementary Figure S1. sNKG2DL plasma levels of individual HNSCC patients. Patients' plasma was analyzed for their sNKG2DL levels by ELISAs (see also Figure 1). **(A)** Cumulative sNKG2DL levels grouped according to treatment naïve patients (primary and relapsed) and patients under palliative therapy or after tumor resection compared to healthy controls. **(B)** HNSCC patients are indicated by number and are presented with increasing disease stage (I-IVC). The mean plasma levels of 12 age-matched healthy controls were used as controls (CO). Bars correspond to mean ± SEM of duplicates or the sum of sNKG2DL for each patient. Statistical significance was assessed by one-way ANOVA.

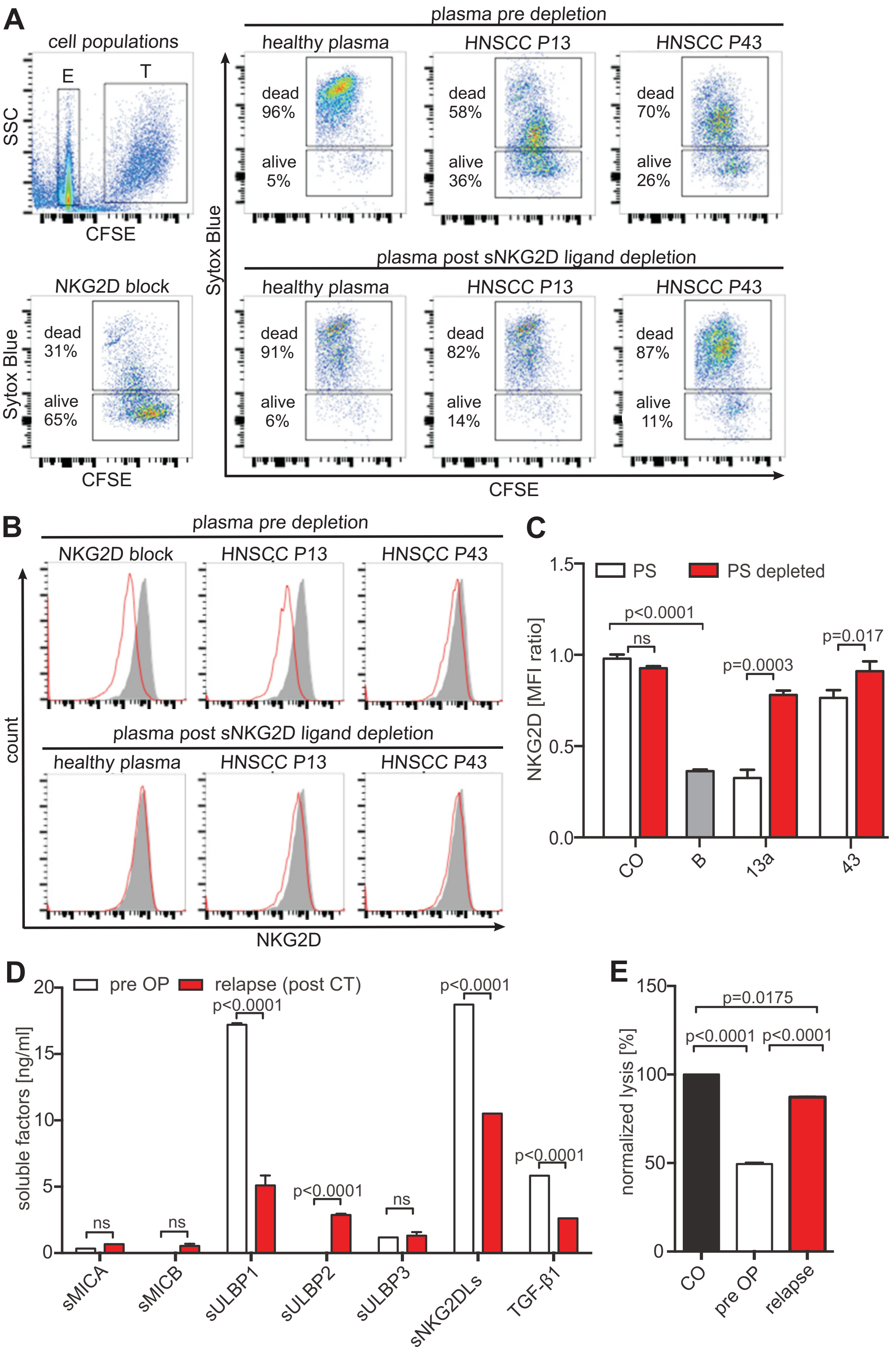

Supplementary Figure S2

Supplementary Figure S2. NKG2D-dependent NK cell inhibition in HNSCC plasma.
(A) Cytotoxicity assays with plasma-treated NK cells. Gating strategy on $CFSE^+$ SiHa target (T) and $CD45^+$ effector NK (E) cells and analysis of live ($CFSE^+$/$SytoxBlue^-$) and dead ($CFSE^+$/$SytoxBlue^+$) target cells. Dot plots of a representative experiment are shown for non-/ and sNKG2DL-depleted healthy plasma and two patients' plasma. **(B)** Flow cytometric analysis of NKG2D surface expression on NK cells treated with plasma from (A). Grey: healthy plasma, red: NKG2D blocking antibody, patients' plasma or depleted plasma. One representative experiment is shown. **(C)** Corresponding NKG2D expression levels represented as mean fluorescence intensity (MFI) ratio normalized to healthy plasma-treated NK cells (CO) as mean ± SEM of duplicates (PS: patients' plasma; B: NKG2D blocking antibody). **(D)** Plasma levels of sNKG2DLs and TGF-β1 of one representative patient at initial diagnosis (pre OP) and relapsed disease presented as mean ± SEM of duplicates. **(E)** Corresponding NK cell cytotoxicity of NK cells pre-treated with HNSCC plasma. Data normalized to healthy control (CO) and bars correspond to mean ± SEM of triplicates. Statistical significance was assessed by unpaired, two-tailed Student's t-test (C) and one-way ANOVA (E), ns= non-significant.

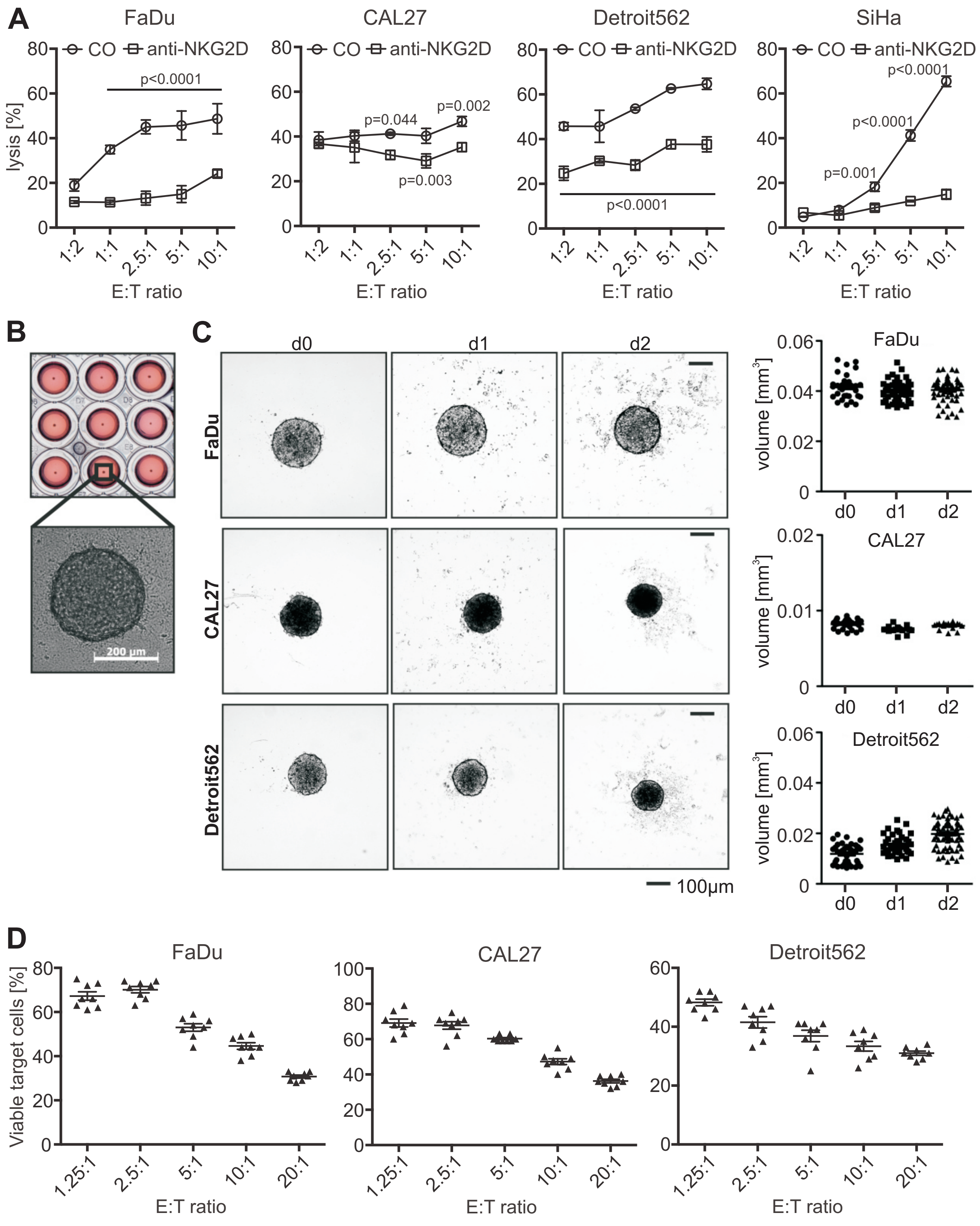

Supplementary Figure S3

Supplementary Figure S3. Establishment of tumor spheroids and NKG2D-dependent NK cell cytotoxicity.

(A) CFSE-labeled FaDu, CAL27, Detroit562 or SiHa cells were co-cultured with primary human NK cells (+/- anti-NKG2D blocking antibody) for 16 h (4 h for SiHa cells) at different E:T ratios and analyzed by flow cytometry. Target cell lysis was calculated by gating on dead ($CFSE^{+}$/$SytoxBlue^{+}$) or viable ($CFSE^{+}$/$SytoxBlue^{-}$) cells. Data are shown as mean ± SEM of triplicates of a representative experiment. **(B)** Representative tumor spheroids are shown in a 96-well plate by transmission microscopy at 50x magnification. **(C)** Growth kinetics of tumor spheroids monitored by phase contrast microscopy at 50x magnification. Representative phase contrast pictures are shown. Size bar corresponds to 100 µm. Tumor spheroid volume (in mm^3) calculated by area determination using Fiji software of individual spheroids from six independent experiments. **(D)** CFSE-labeled tumor spheroids were co-cultured with NK cells at different E:T ratios for 48 h. Tumor spheroid destruction was quantified by flow cytometry and evaluation of viable ($CFSE^{+}$/$SytoxBlue^{-}$) and dead ($CFSE^{+}$/$SytoxBlue^{+}$) target cells. Data is presented as percentage viable target cells of 8 individual tumor spheroids of a representative experiment. Statistical significance was assessed by one-way ANOVA.

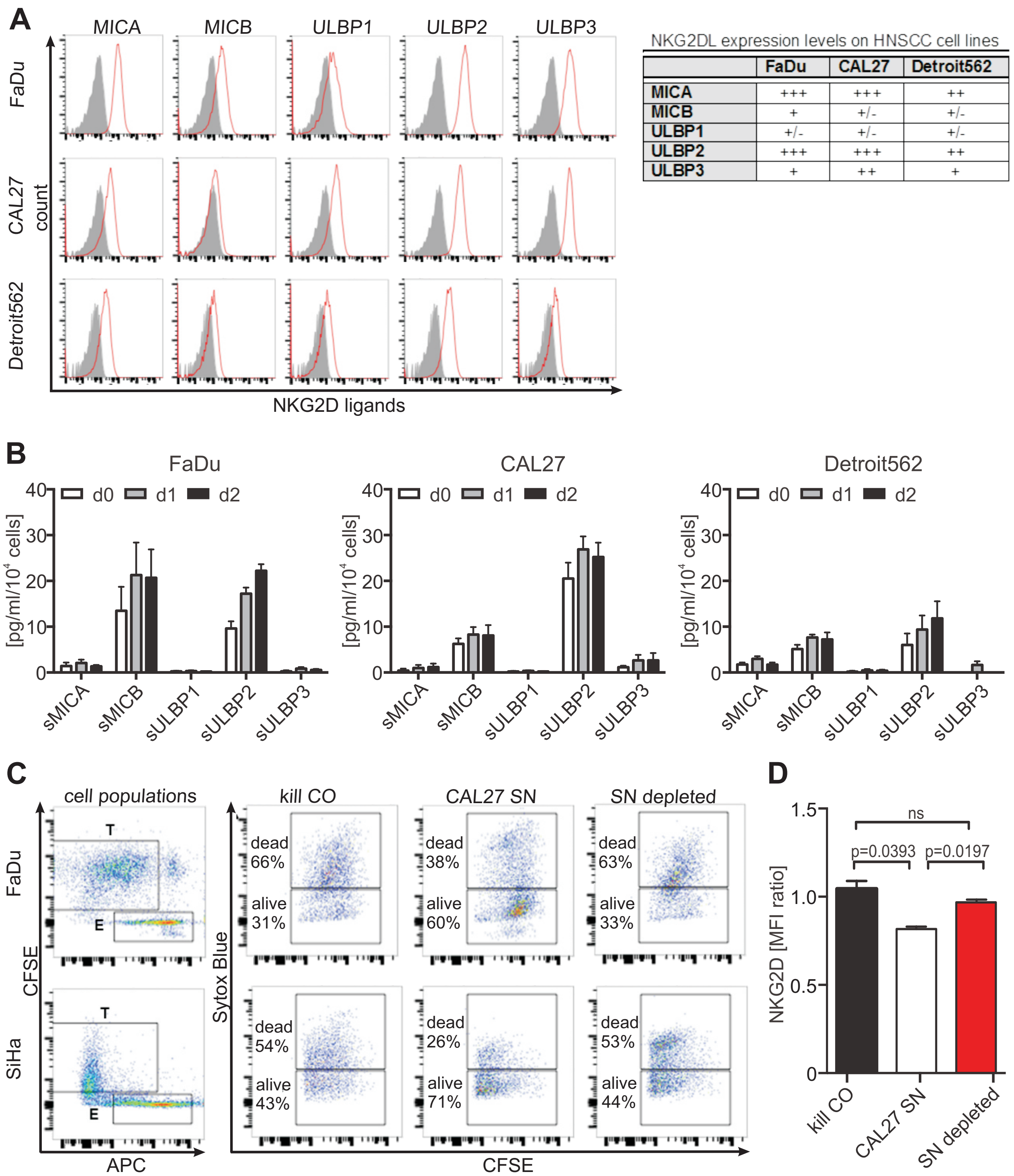

NKG2DL expression levels on HNSCC cell lines

	FaDu	CAL27	Detroit562
**MICA**	+++	+++	++
**MICB**	+	+/-	+/-
**ULBP1**	+/-	+/-	+/-
**ULBP2**	+++	+++	++
**ULBP3**	+	++	+

Supplementary Figure S4

Supplementary Figure S4. Expression and shedding of NKG2DLs in HNSCC tumor spheroids.

(A) Surface expression of NKG2DLs (MICA, MICB, ULBP1-3) on FaDu, CAL27 and Detroit562 tumor spheroids analyzed by flow cytometry. Grey: isotype control, red: NKG2DL antibody. One representative experiment is shown. Expression patterns were calculated from mean fluorescence intensity (MFI) and presented as +/- = no/weak; + = weak, ++ =moderate; +++ =high expression and depicted in the table. **(B)** Tumor spheroid supernatants were collected on three consecutive days (d0 – d2) and concentrated 10-fold. Shedding of sMICA/B, sULBP1-3 was quantified by ELISAs. Data are shown as mean ± SEM in pg/ml/10^4 cells of three independent experiments, measured in duplicates. **(C)** Tumor spheroid cytotoxicity assays with treated NK cells (CAL27 SN or SN depleted). Gating strategy on CFSE-labeled target (T) and CD45-stained effector NK (E) cells and analysis of live ($CFSE^+$/$SytoxBlue^-$) and dead ($CFSE^+$/$SytoxBlue^+$) target cells. Dot plots of one representative experiment are shown for FaDu and SiHa spheroids. **(D)** Corresponding NKG2D surface expression of CAL27 SN/depleted SN treated NK cells. Data is represented as MFI ratio normalized to kill CO as mean ± SEM of duplicates of a representative experiment. Statistical significance was assessed by one-way ANOVA, ns = non-significant.

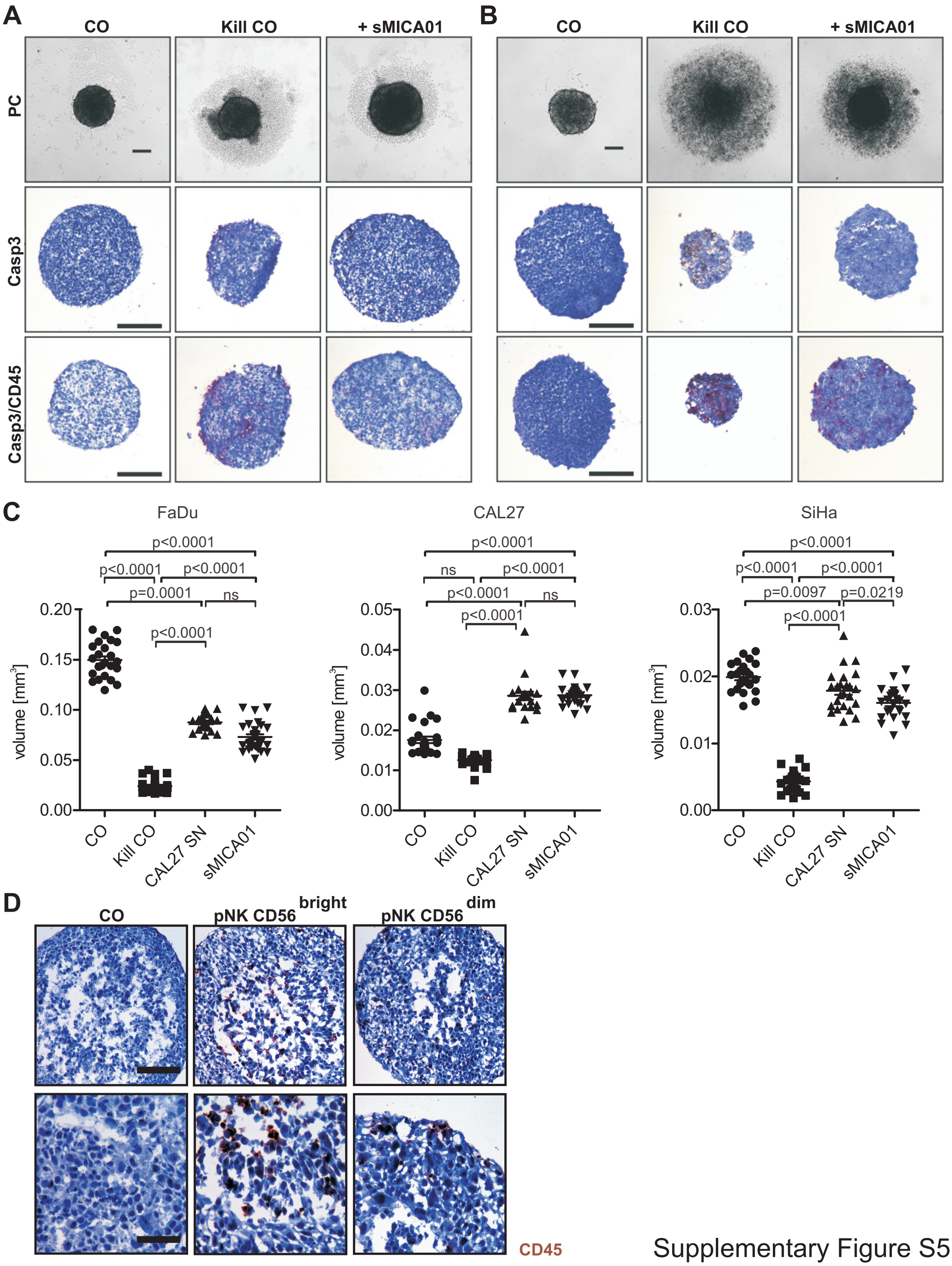

Supplementary Figure S5

Supplementary Figure S5. NK cell cytotoxicity an infiltration into tumor spheroids.

NK cells were pre-treated overnight with CAL27 supernatant, sMICA01 or were left untreated (kill CO) prior to co-culturing with FaDu, CAL27 or SiHa cells. Tumor spheroid formation was monitored by light microscopy for 48 h. Representative phase contrast pictures of CAL27 **(A)** and SiHa **(B)** tumor spheroids at 50x magnification are shown (size bar = 100 µm). Cryosections of spheroids after 48 h were stained for NK cells (anti-CD45 antibody, red) and apoptosis (anti-active caspase-3 antibody, brown). Representative histology pictures are shown at 200x magnification (size bar = 200 µm). **(C)** Data analysis of NK cell cytotoxicity in FaDu, CAL27 and SiHa tumor spheroids. Residual tumor spheroid size is plotted as the volume of individual spheroids. Data are shown as mean ± SEM. Spheroid volume (in mm^3) was calculated based on phase contrast image analysis by area determination using Fiji software. Statistical significance was assessed by unpaired, two-tailed Student's t-test, ns = non-significant. **(D)** FaDu tumor spheroids co-cultured with sorted NK cells (cytotoxic $CD56^{dim}/CD16^{+}$ and regulatory $CD56^{bright}/CD16^{-}$). Cryosections of tumor spheroids were stained for NK cell infiltration (anti-CD45 antibody, red). Representative pictures are shown under 200x (size bar = 200 µm) and 400x (size bar = 100 µm) magnification.

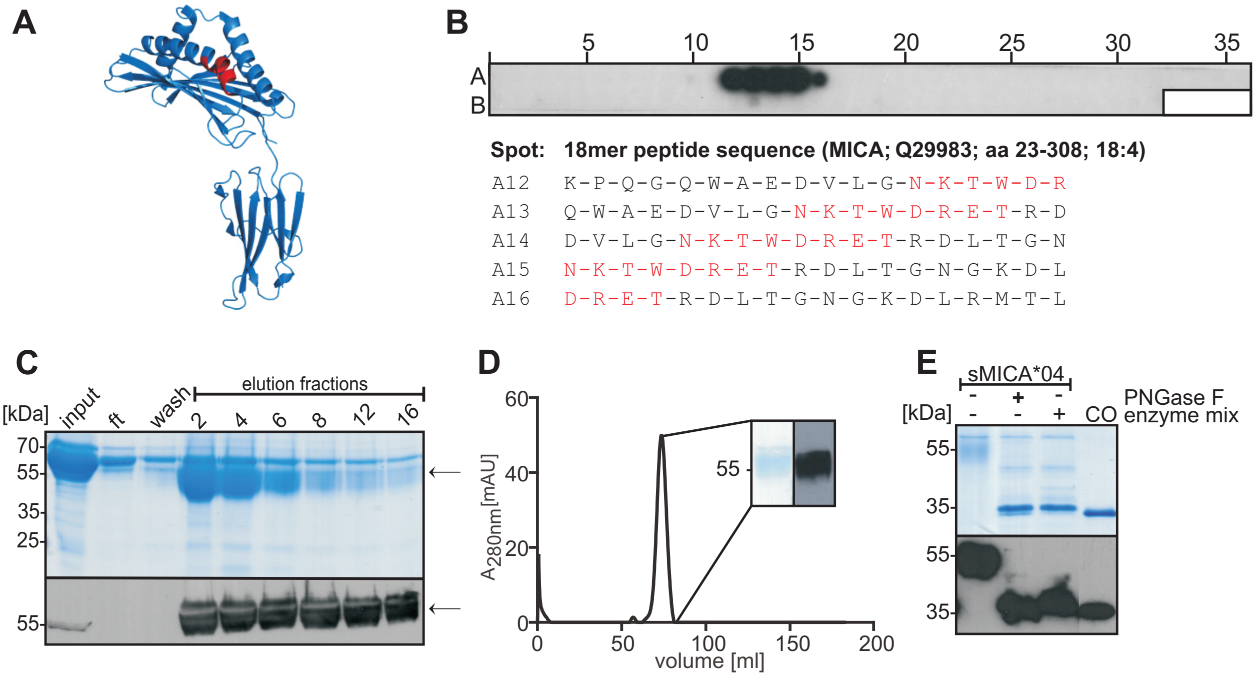

F

```
MICA_HUMAN      MGLGPVFLLLAGIFPFAPPGAAAEPHSLRYNLTVLSWDGSVQSGFLTEVHLDGQPFL
MIC1_MACMU              F--S-----R------L------V----R---------A-------L--
MIC2_MACMU   RVLLFLGRVLLF--S--S-TR-------------V----R------E--A-G-----LFV

MICA_HUMAN   RCDRQKCRAKPQGQWAEDVLGNKTWDRETRDLTGNGKDLRMTLAHIKDQKEGLHSLQEIR
MIC1_MACMU   LY-------R---E-S-----A----T--G---E-------------G--G--------K
MIC2_MACMU   -Y--ETR--R-------A---D    Q--E---E-AQ---R--T--EG--G------K-K

MICA_HUMAN   VCEIHEDNSTRSSQHFYYDGELFLSQNLETKEWTMPQSSRAQTLAMNVRNFLKEDAMKTK
MIC1_MACMU   ----------GGLR----------------Q---EL---------L-I---W---T----
MIC2_MACMU   I---Y--G--GGFW-------R---L----QK--VA----------   --W---T---N


MICA_HUMAN   THYHAMHADCLQELRRYLKSGVVLRRTVPPMVNVTRSEASEGNITVTCRASGFYPWNITL
MIC1_MACMU   ---RTVQ----KK-QQ--E-R-AV---A-------H-------------------R--A-
MIC2_MACMU   ---R--R----KK----Q--R-AV-----------HG------------------G----

MICA_HUMAN   SWRQDGVSLSHDTQQWGDVLPDGNGTYQTWVATRICQGEEQRFTCYMEHSGNHSTHPVPS
MIC1_MACMU   T--------N-NA----GI---Q------------R-------A----------------
MIC2_MACMU   T-----------A---------R----Y-------R-------A-----------Y----

MICA_HUMAN   GKVLVLQSHWQTFHVS    AVAAAAIFVIIIFYVRCCKKKTSAAEGPELVSLQVLDQHP
MIC1_MACMU   -----F--Q-LDIPYVLAVA-A----AIFV--L--L-----------------RT-----
MIC2_MACMU   --PP----ERLNLLYVPV---AV-VV-AF---CVH--K----------------T-----

MICA_HUMAN   VGTSDHRDATQLGFQPLMSDLGSTGSTEGA
MIC1_MACMU   ---G---
MIC2_MACMU   ---G---------S-----AP-----
```

G

```
NKG2D_HUMAN  MGWIRGRRSRHSWEMSEFHNYNLDLKKSDFSTRWQKQRCPVVKSKCRENASPFFFCCFIA
NKG2D_MACMU  --------P--NL--------K-G-A-------C-------I---------L--------

NKG2D_HUMAN  VAMGIRFIIMVAIWSAVFLNSLFNQEVQIPLTESYCGPCPKNWICYKNNCYQFFDESKNW
NKG2D_MACMU  -----------T------------------------------------------N-----

NKG2D_HUMAN  YESQASCMSQNASLLKVYSKEDQDLLKLVKSYHWMGLVHIPTNGSWQWEDGSILSPNLLT
NKG2D_MACMU  ------------------------------------------------------------

NKG2D_HUMAN  IIEMQKGDCALYASSFKGYIENCSTPNTYICMQRTV
NKG2D_MACMU  ------------------------I-----------
```

Supplementary Figure S6

Supplementary Figure S6. Epitope mapping of the anti-MICA antibody and purification of sMICA*04.

(A) Crystal structure of the sMICA ectodomain (grey; aa 23-297) with the anti-MICA antibody epitope (NKTWDRET; red) in the α1-helical domain; PDB 1HYR. **(B)** Peptide spot arrays of human MICA incubated with the anti-MICA antibody. The signals of spots A11-A16 mark the epitope region and corresponding sequences are depicted. The minimal required epitope comprises amino acids NKTWDRET (red). **(C)** Reducing SDS-PAGE (Coomassie-stained) and immunoblot of aliquots (input, flow-through (ft), wash and eluate fractions) of sMICA*04 after affinity purification from HEK293T cell culture supernatants. **(D)** Size exclusion chromatography (SEC) of the purified sMICA*04 on a Superdex 200 column. Pooled peak fractions were analyzed by Coomassie-stained SDS-PAGE and immunoblot. **(E)** Deglycosylation of the purified sMICA*04. Reducing SDS-PAGE (Coomassie-stained) and immunoblot of untreated (lane 1), PNGaseF treated (lane 2) or enzyme mix treated (lane 3) sMICA*04. Non-glycosylated sMICA*04 produced in *E.coli* served as control (CO; lane 4). **(F)** Sequence alignment of human MICA (UniProtKB Q29983, MICA_HUMAN) and the *Macaca mulatta* MIC homologous MIC1 and MIC3 (UniProtKB O98268, MIC1_MACMU; Q9XS20, MIC3_MACMU). Dash: identical amino acids, grey: MICA signal peptide sequence, bold blue: N-linked glycosylation sites, red box: anti-MICA antibody epitope. **(G)** Sequence alignment of human NKG2D (UniProtKB P26718, NKG2D_HUMAN) and the *Macaca mulatta* homologous (UniProtKB Q9MZJ7, NKG2D_MACMU).

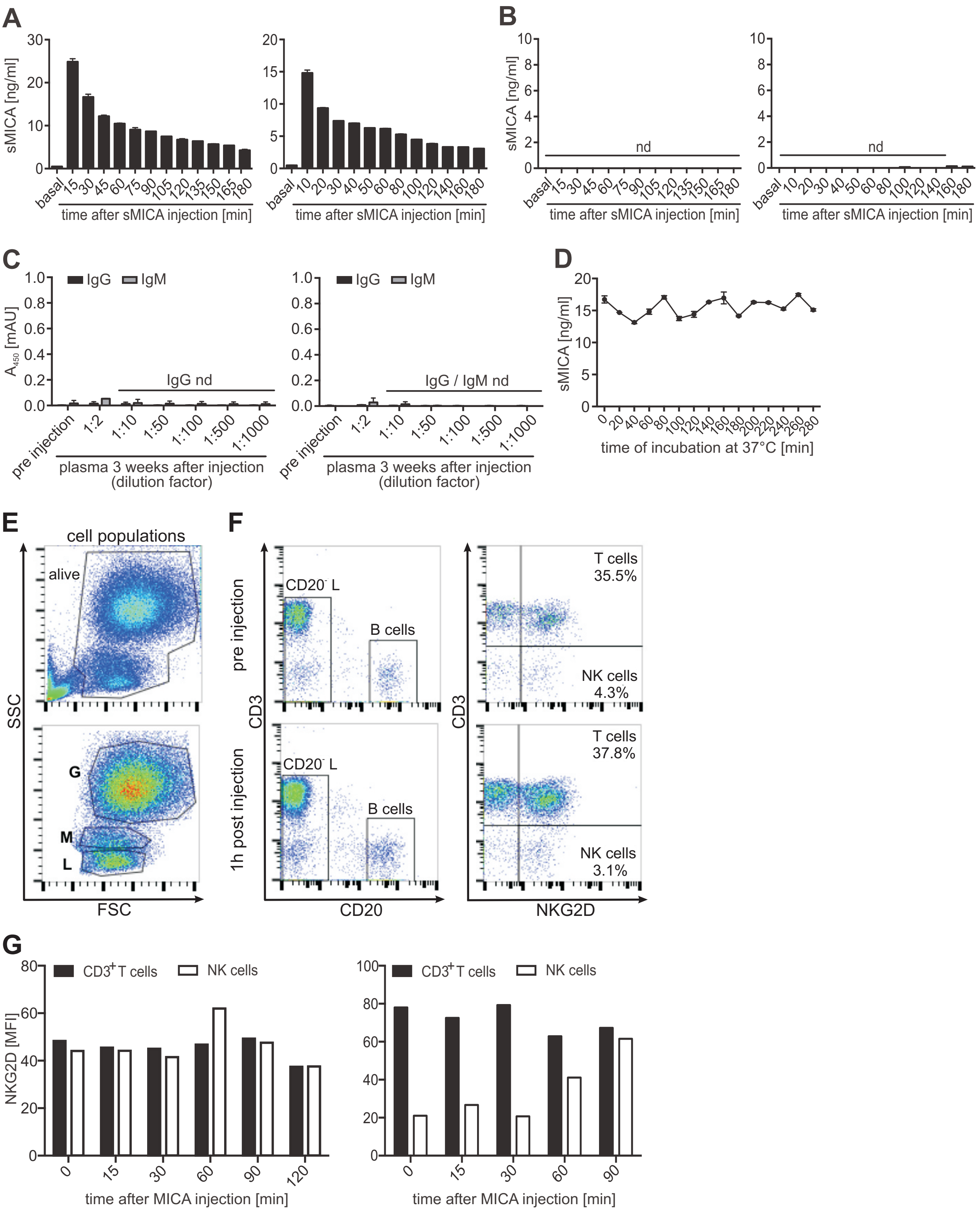

Supplementary Figure S7

Supplementary Figure S7. Analysis of sMICA body distribution, plasma stability and NKG2D expression levels in rhesus monkeys.

For the pilot study, two animals (left/right) were injected with 100µg/l blood volume sMICA*04. The sMICA plasma and urine concentrations were determined before (basal) and after injection (over three hours) by ELISA in duplicates. **(A)** Analysis of sMICA*04 plasma levels to evaluate sMICA body distribution. Data is represented as mean ± SEM. **(B)** Analysis of sMICA*04 in rhesus macaque urine to determine renal clearance of injected sMICA*04. **(C)** Determination of anti-MICA IgG/IgM monkey antibodies in the plasma three weeks after sMICA*04 injection. Plasma dilutions of two animals were probed for anti-MICA IgG (black bars) or IgM (grey bars) antibodies on sMICA*04 coated ELISA plates. Data is represented as mean ± SEM. **(D)** Rhesus macaque plasma was supplemented with sMICA*04 and incubated at 37°C to analyze sMICA plasma stability. Plasma samples were measured every 20 min in duplicates by ELISA. Data is represented as mean ± SEM. (**E**) Flow cytometric analysis of primary rhesus PBMCs from whole blood samples during apheresis. Gating strategy for living (alive) cells and determination of granulocyte (G), monocyte (M) and lymphocyte (L) populations (left). Analysis of T cells ($CD3^+$/$NKG2D^+$) and NK cells ($CD3^-$/$NKG2D^+$) from the $CD20^-$ lymphocyte population (right). Representative dot plots are shown for PBMCs of one animal at time of sMICA*04 injection and during apheresis (1h post injection). **(F)** Flow cytometric analysis of NKG2D surface expression on $CD3^+$ T cells and $CD3^-$ NK cell during apheresis for both animals.
